# Supplementary material for: The importance of degradation mode analysis in parameterising lifetime prediction models of lithium-ion battery degradation
Source: Nat Commun. 2025 Mar 21;16:2776. doi: 10.1038/s41467-025-57968-3 (PMC11926349; doi:10.1038/s41467-025-57968-3)
Supplement: Supplementary file 1 — Supplementary Information [file 41467_2025_57968_MOESM1_ESM.pdf]

Supplementary information

The importance of degradation mode analysis in parameterizing lifetime prediction models of lithium-ion battery degradation

Ruihe Li[1,2], Niall D. Kirkaldy[1,2], Fabian F. Oehler[3], Monica Marinescu[1,2], Gregory J. Offer[1,2,*], Simon E. J. O'Kane[1,2]

[1]Department of Mechanical Engineering, Imperial College London, UK

[2]The Faraday Institution, UK

[3]Technical University of Munich (TUM), School of Engineering and Design, Department of Energy and Process Engineering, Institute for Electrical Energy Storage Technology (EES), Arcisstr. 21, 80333 Munich, Germany

* Corresponding author: Gregory J. Offer, gregory.offer@imperial.ac.uk

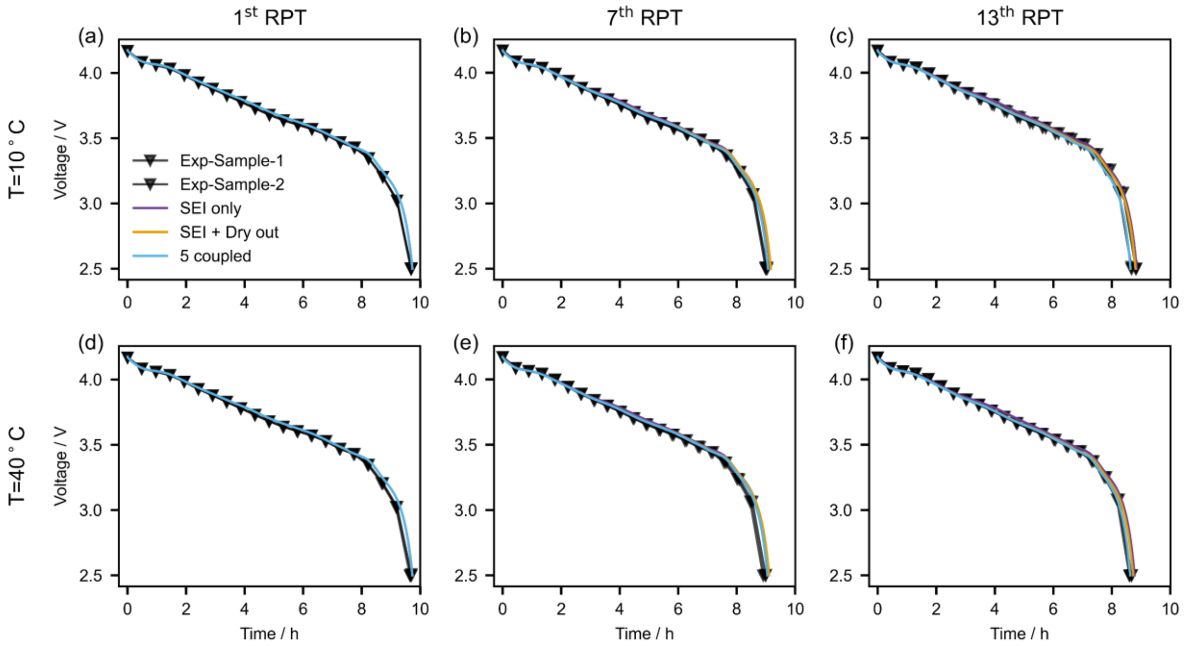

Supplementary Figure 1. C/10 discharge voltage curves at 10°C (a ~ c) and 40°C (d ~ f) for Experiment 2. Note that all RPT are carried out at 25°C, the temperature just denotes their ageing temperature. Source data for this figure are provided as a Source Data file.

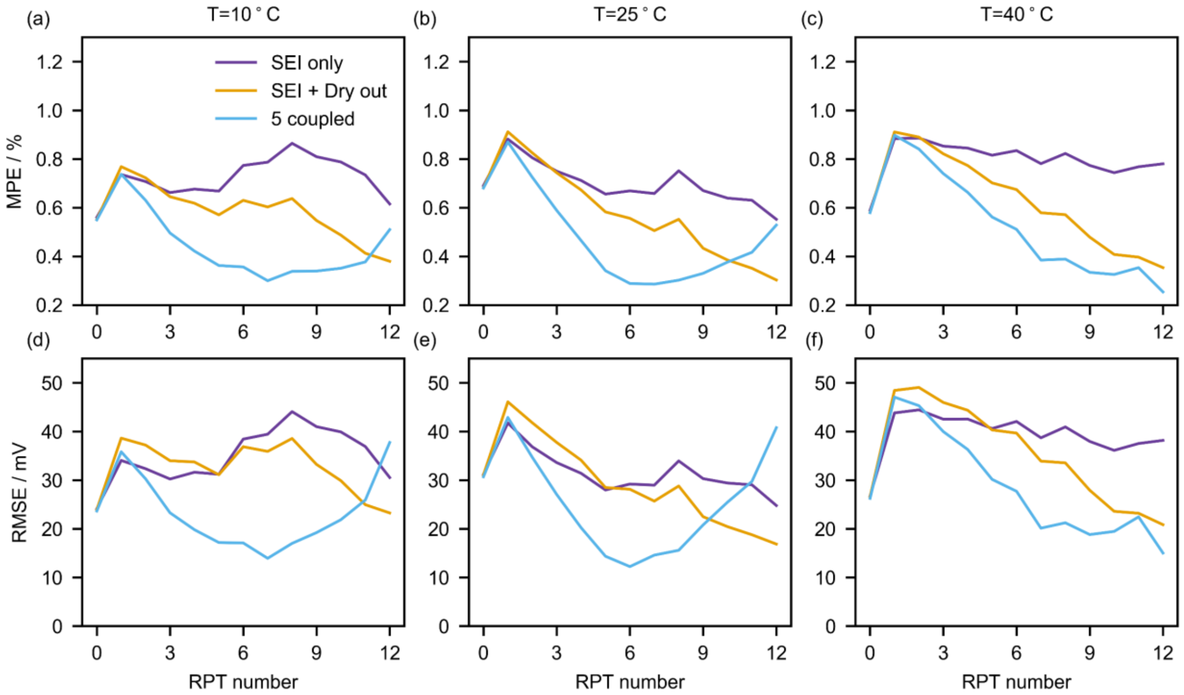

Supplementary Figure 2. MPE (a ~ c) and RMSE (d ~ e) of C/10 voltage during RPTs for Experiment 2. Source data for this figure are provided as a Source Data file.

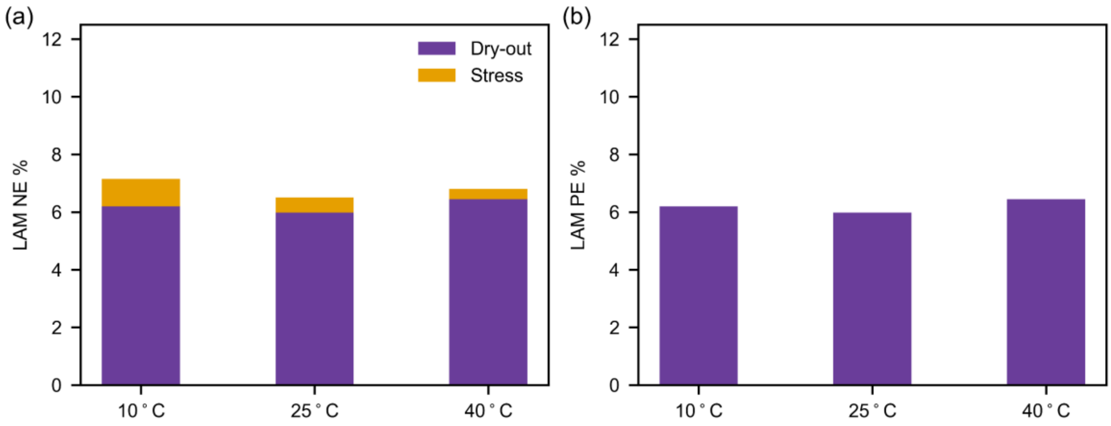

Supplementary Figure 3. Different contributions to LAM in the 5 coupled model at three temperatures for Experiment 2. (a) shows the LAM at the negative electrode (NE) while (b) shows the LAM at the positive electrode (PE). Source data for this figure are provided as a Source Data file.

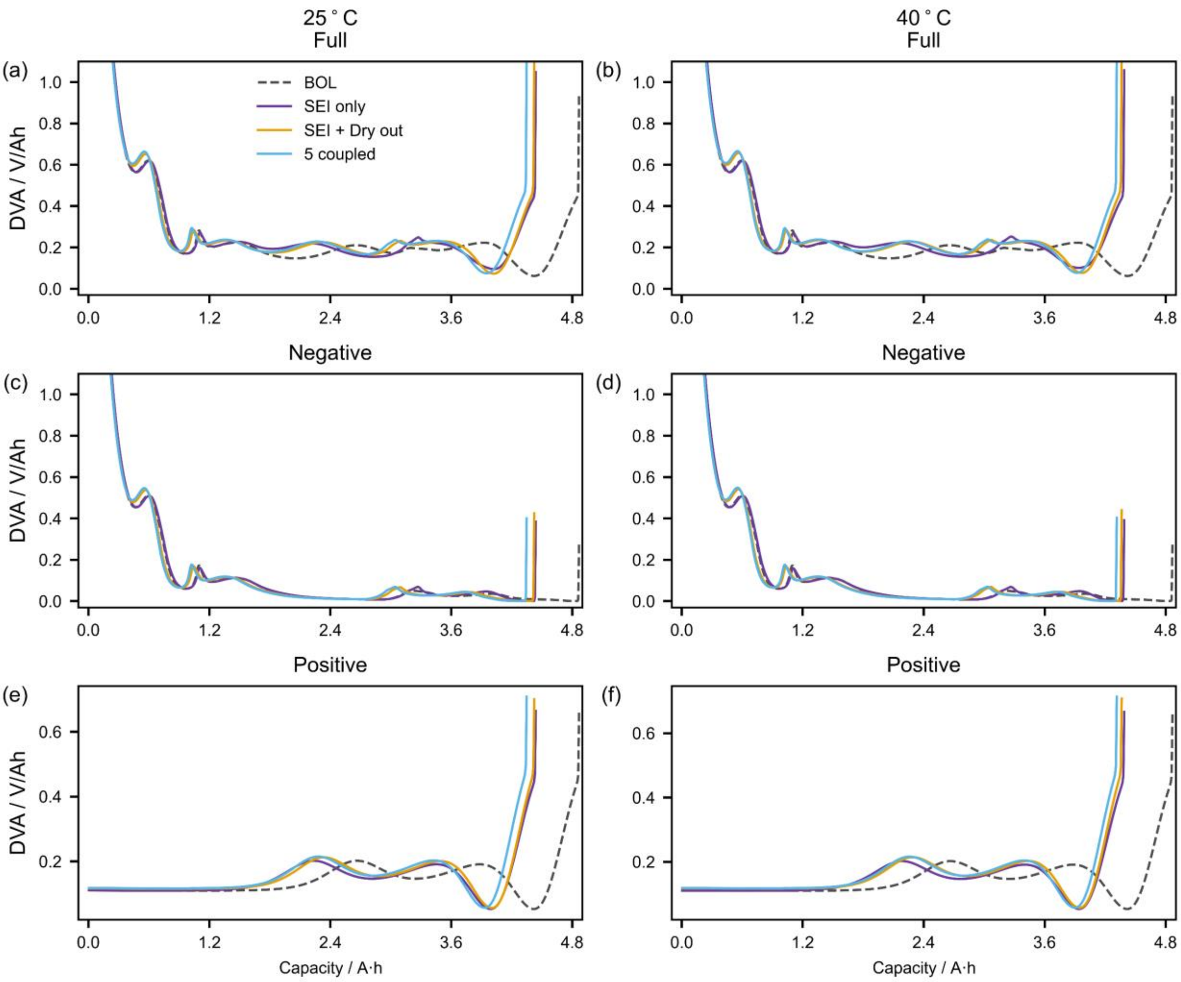

Supplementary Figure 4. DVA curves of C/10 discharge for Experiment 2. Dash lines refer to BOL and solid line refers to the 11th RPT for different models). (a ~ c) refer to cells aged at 25°C and (d ~ f) refer to cells aged at 40°C. Source data for this figure are provided as a Source Data file.

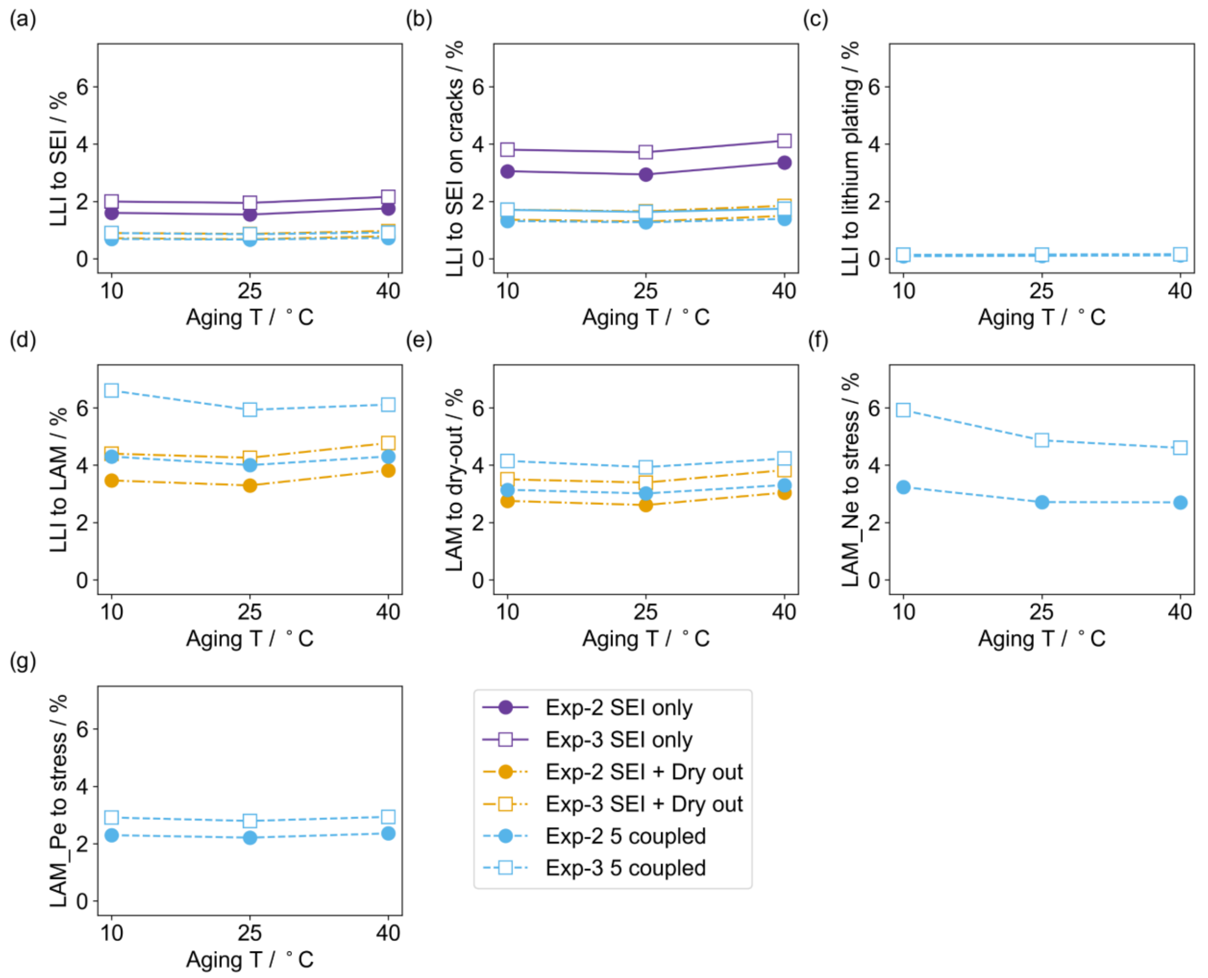

Supplementary Figure 5. Contributions of different degradation mechanisms on DMs for the three models. These contributions are: (a) LLI due to SEI, (b) LLI due to SEI on cracks, (c) LLI due to lithium plating, (d) LLI due to LAM, (e) LLI due to electrolyte dry-out, (f) LAM at the negative electrode (NE) due to mechanical stress, (g) LAM at the positive electrode (PE) due to mechanical stress. Source data for this figure are provided as a Source Data file.

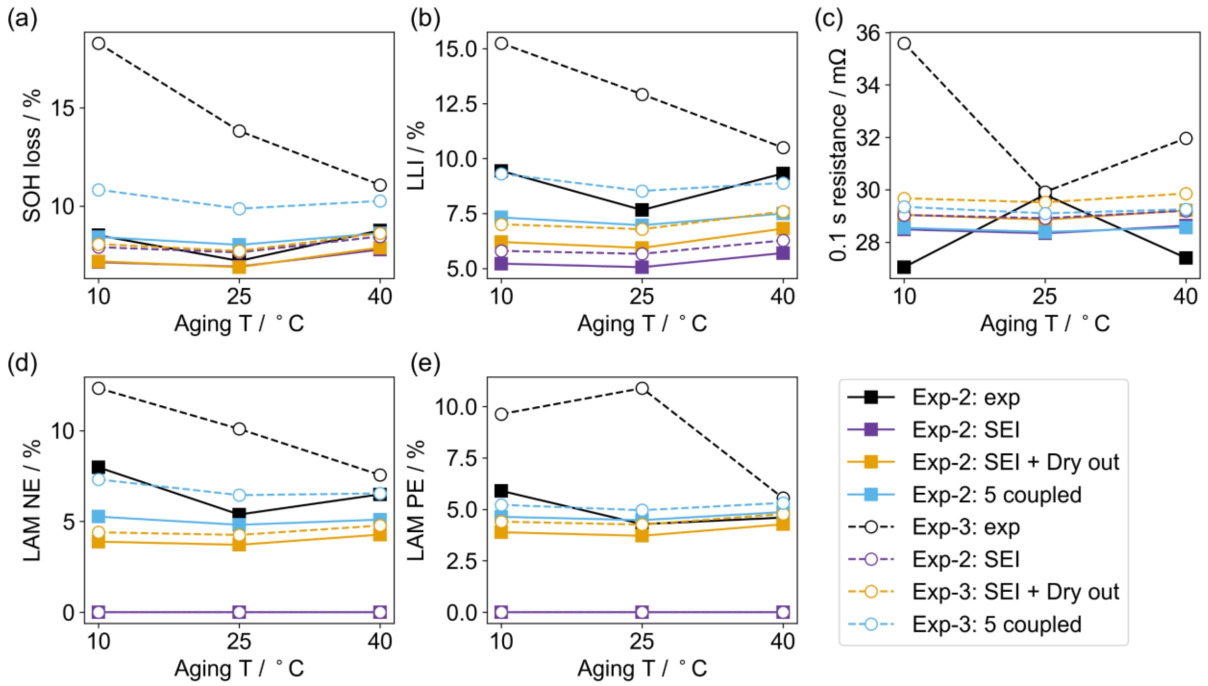

Supplementary Figure 6. Direct comparison between modelling and experimental data for Experiment 2 and Experiment 3 at the charge throughput of 6.37 kAh. (a) SOH loss, (b) LLI, (c) 0.1s resistance, (d) LAM at the negative electrode (NE), (e) LAM at the positive electrode (PE). Source data for this figure are provided as a Source Data file.

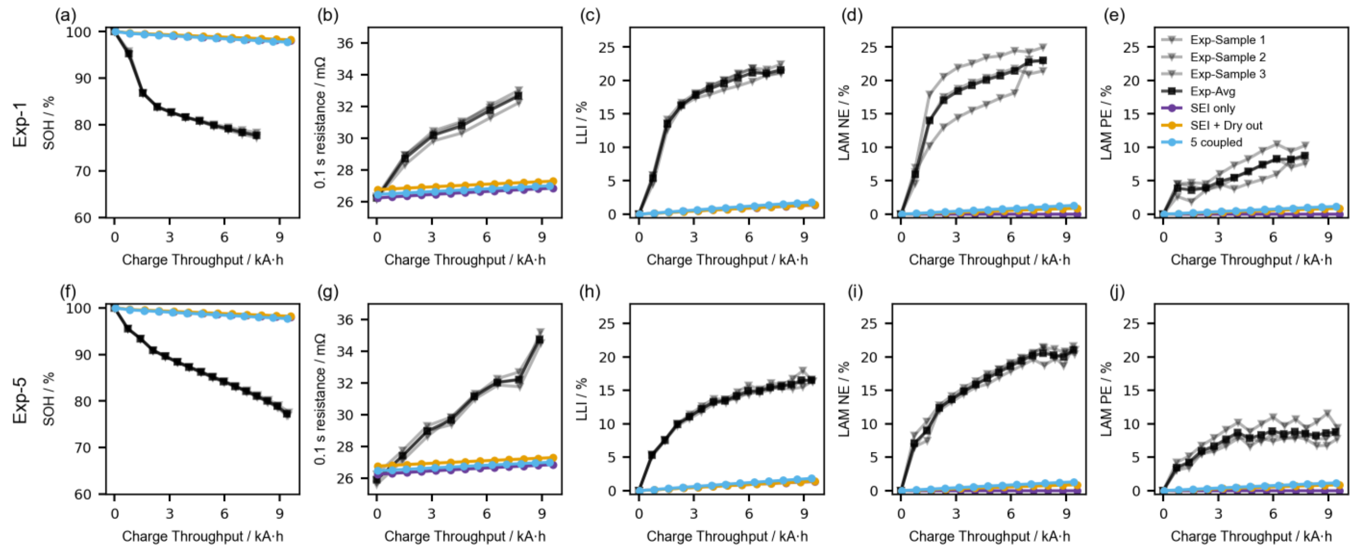

Supplementary Figure 7. Model validation against Experiment 1 and Experiment 5 at 40°C. All three models fail to predict both experiments. (a ~ e) refer to the validation results on Experiment 1 and (f ~ j) refer to the validation results on Experiment 5. Source data for this figure are provided as a Source Data file.

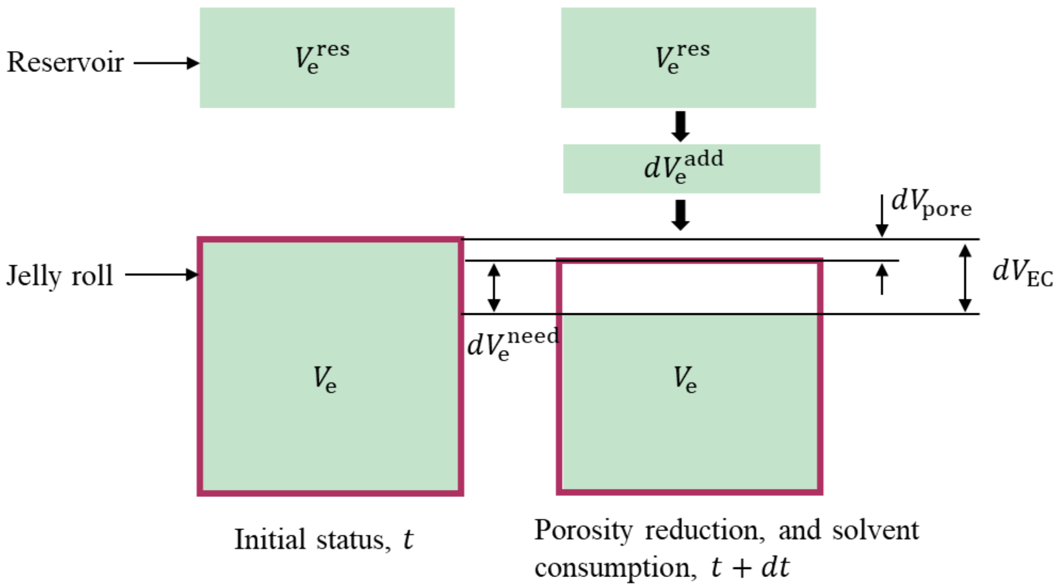

Supplementary Figure 8. Illustration of different types of volumes in the solvent consumption model. The red frame represents the total pore volume in the jelly roll (V_{pore}). The green block inside the red frame represents the electrolyte volume inside the jelly roll (V_e). The green block outside the red frame represents the electrolyte in the reservoir (V_e^{res}). From t to $t + dt$, the total pore volume inside the jelly roll reduces (dV_{pore}) due to deposit SEI layer growth and lithium plating, the electrolyte inside the jelly roll reduces due to EC consumption (dV_{EC}). The sizes of dV_e^{need} and V_e^{res} in this figure stands for Case-1 in Supplementary Note 6, i.e., certain amount of electrolyte (dV_e^{add}) will replenish from the reservoir to the jelly roll to completely fill the gap left by dV_e^{need}.

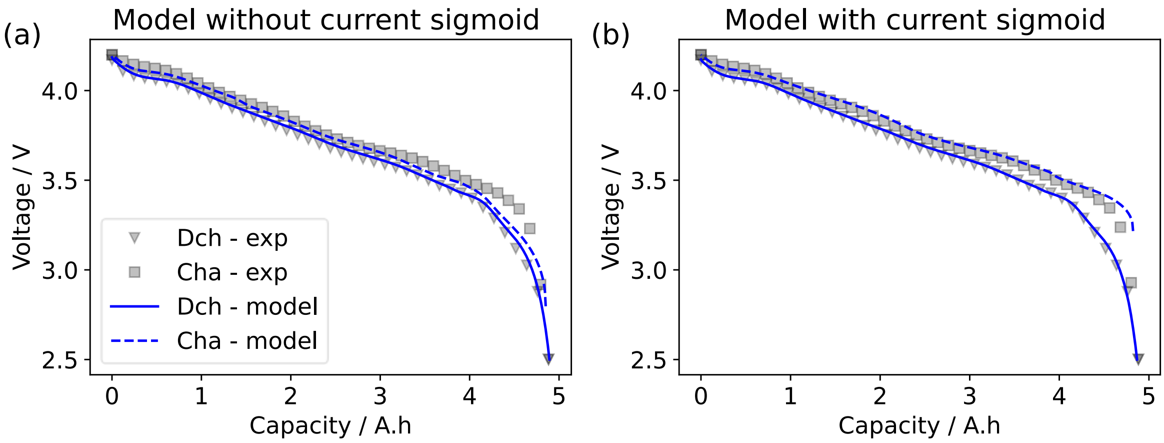

Supplementary Figure 9. Comparisons between the modelling and experimental 0.1C charge/discharge voltage curves (a) without and (b) with current sigmoid. Source data for this figure are provided as a Source Data file.

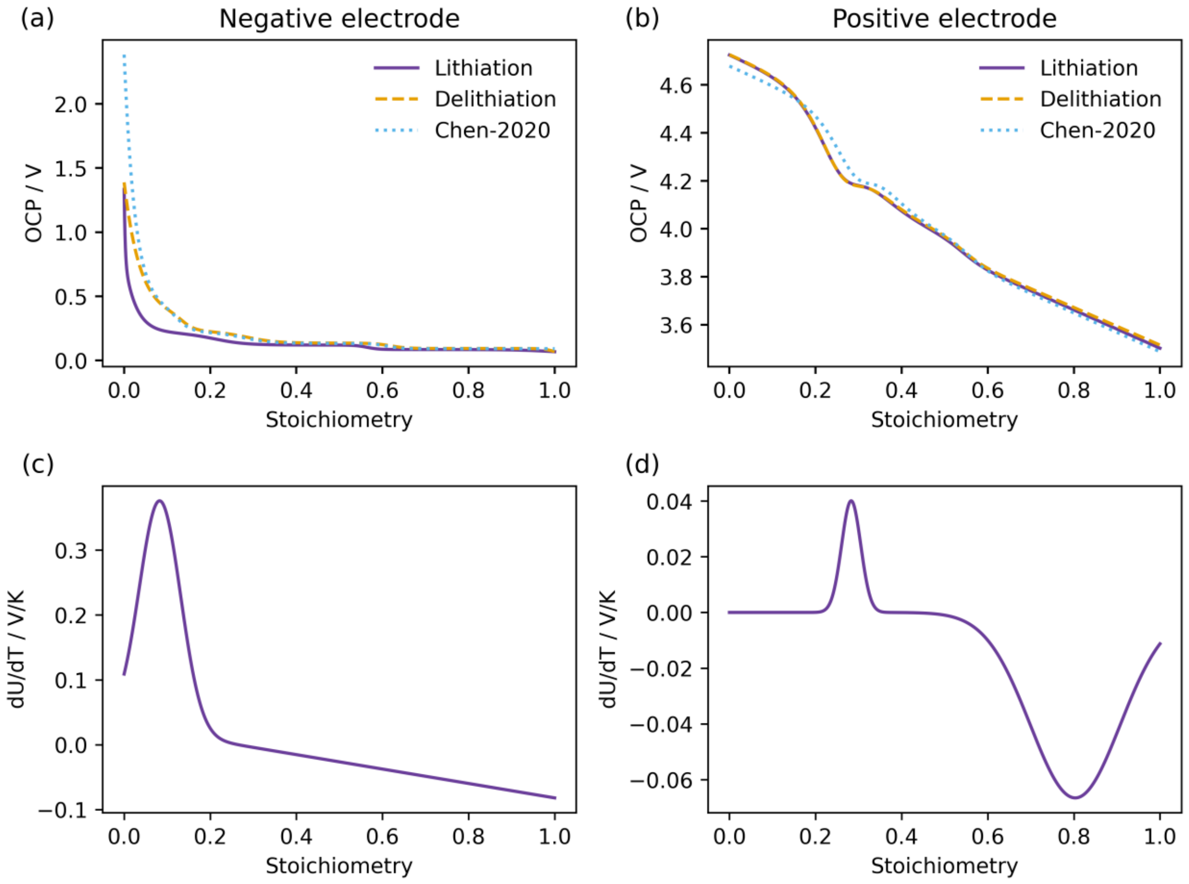

Supplementary Figure 10. Open-circuit potential (OCP) and entropic change[1] of both electrodes. (a) OCP of the negative electrode, (b) OCP of the positive electrode, (c) entropic change of the negative electrode, (d) entropic change of the positive electrode. Source data for this figure are provided as a Source Data file.

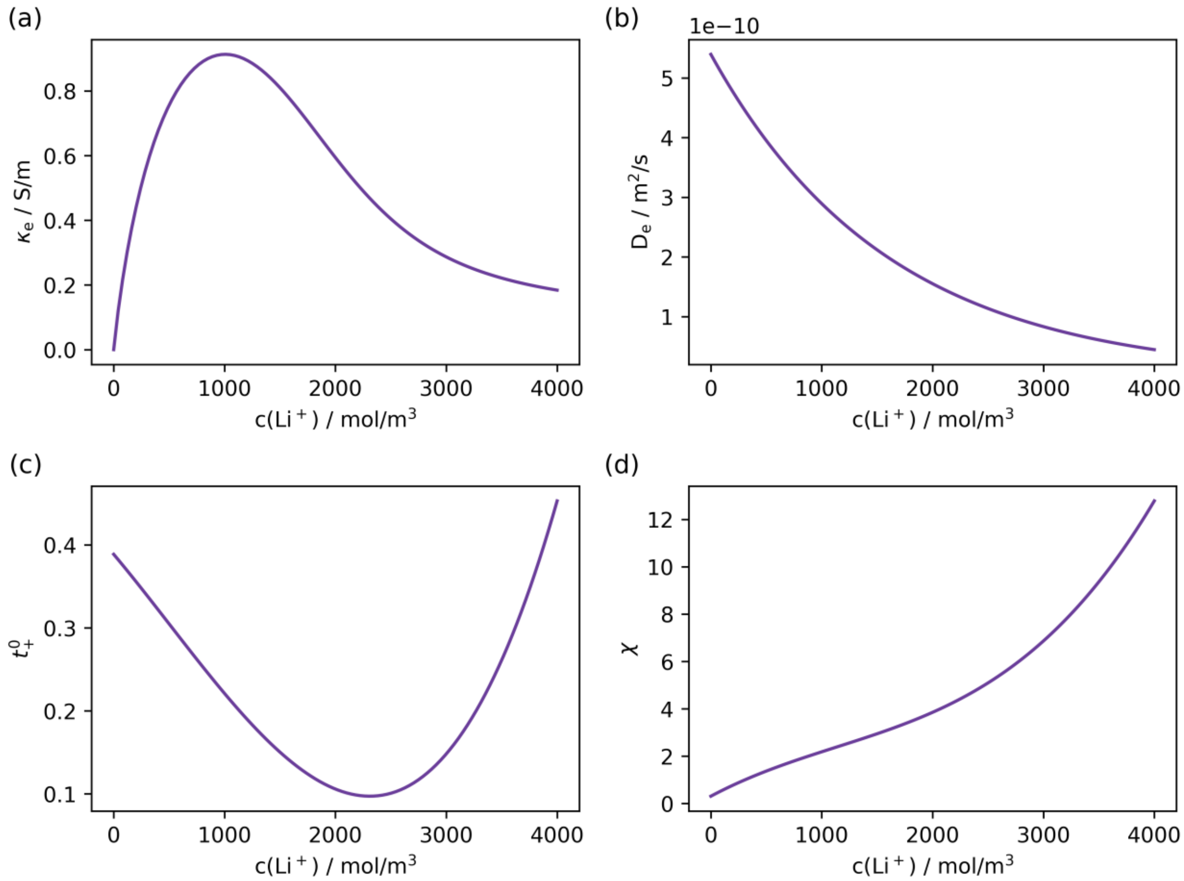

Supplementary Figure 11. Electrolyte parameters used in this work. These include (a) electrolyte conductivity, (b) diffusivity, (c) cation transference number and (d) thermodynamic factor for EC:EMC 3:7 wt% in $LiPF_6$[2] at 25°C. Source data for this figure are provided as a Source Data file.

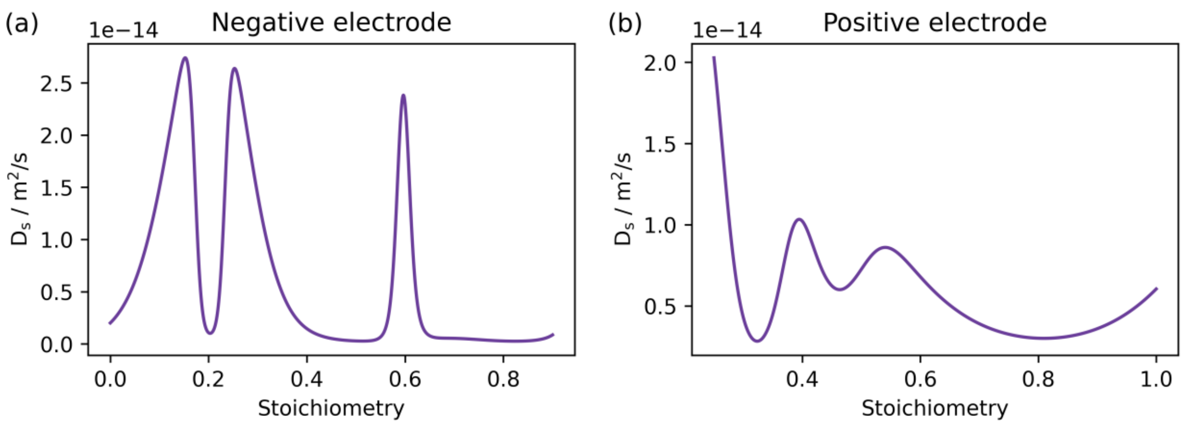

Supplementary Figure 12. Solid-phase diffusivity at 25 °C used in this work[1]. These include the diffusivities for (a) the negative and (b) positive electrodes. Source data for this figure are provided as a Source Data file.

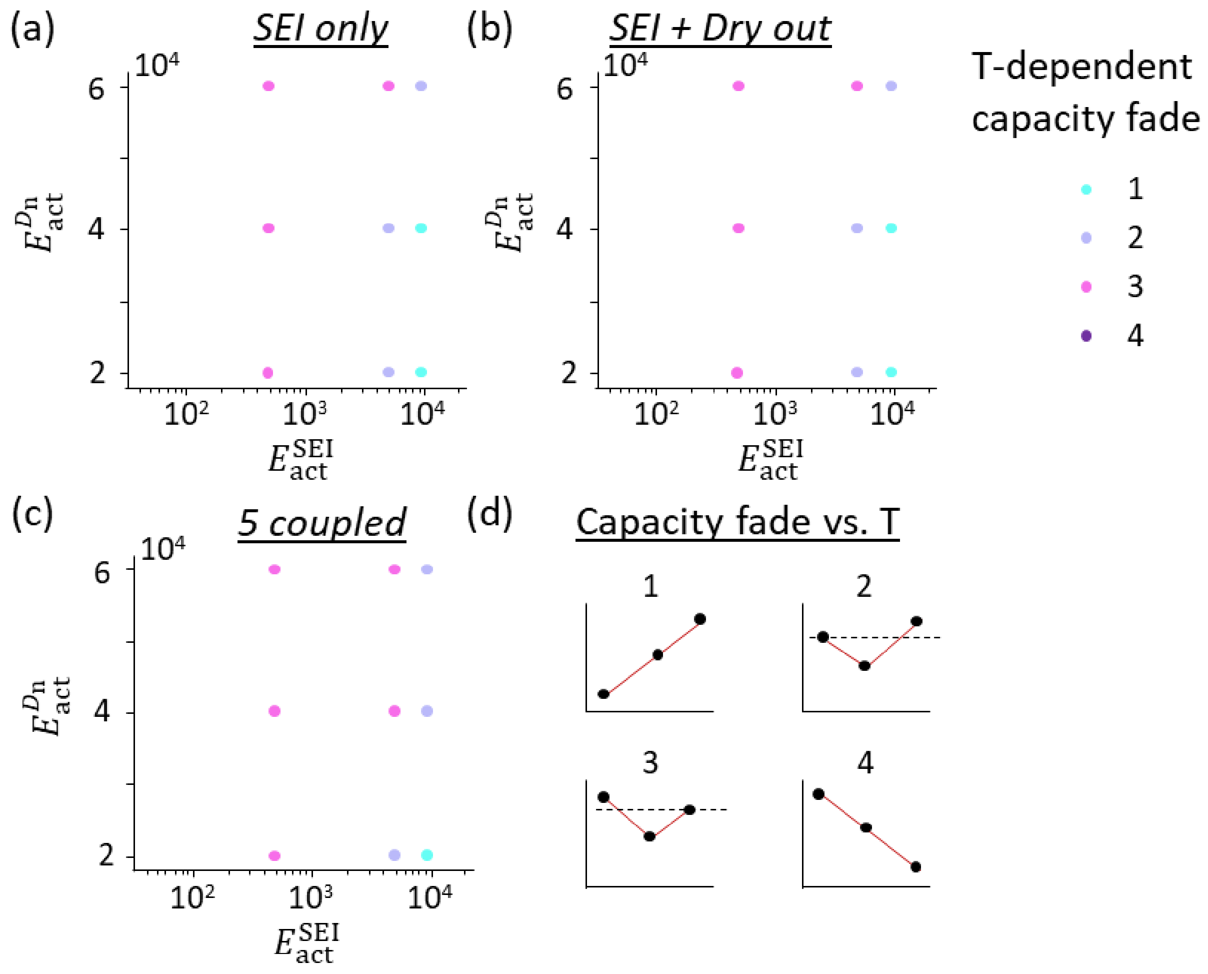

Supplementary Figure 13. Temperature dependent capacity fade affected by E_{act}^{SEI} and $E_{act}^{D_{s,n}}$. Note that the "4" type is not captured in all three models under the 9 combinations of these two parameters for Experiment 2. (a) shows the results for the SEI only model, (b) the SEI +Dry-out model, and (c) the 5 coupled model. (d) describes the four types of temperature dependency. Source data for this figure are provided as a Source Data file.

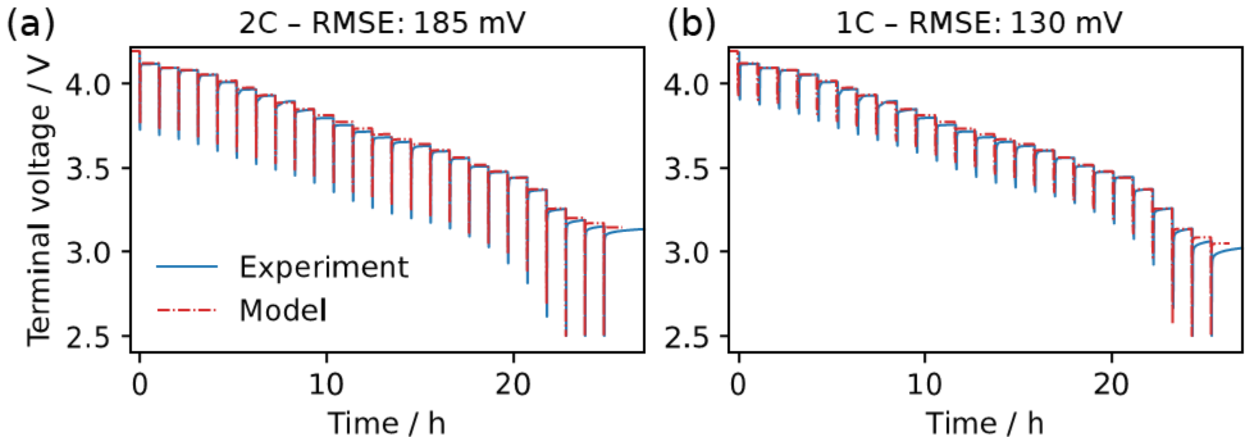

Supplementary Figure 14. Validation against GITT during the first RPT. These include the results at (a) 2C and (b) 1C. Source data for this figure are provided as a Source Data file.

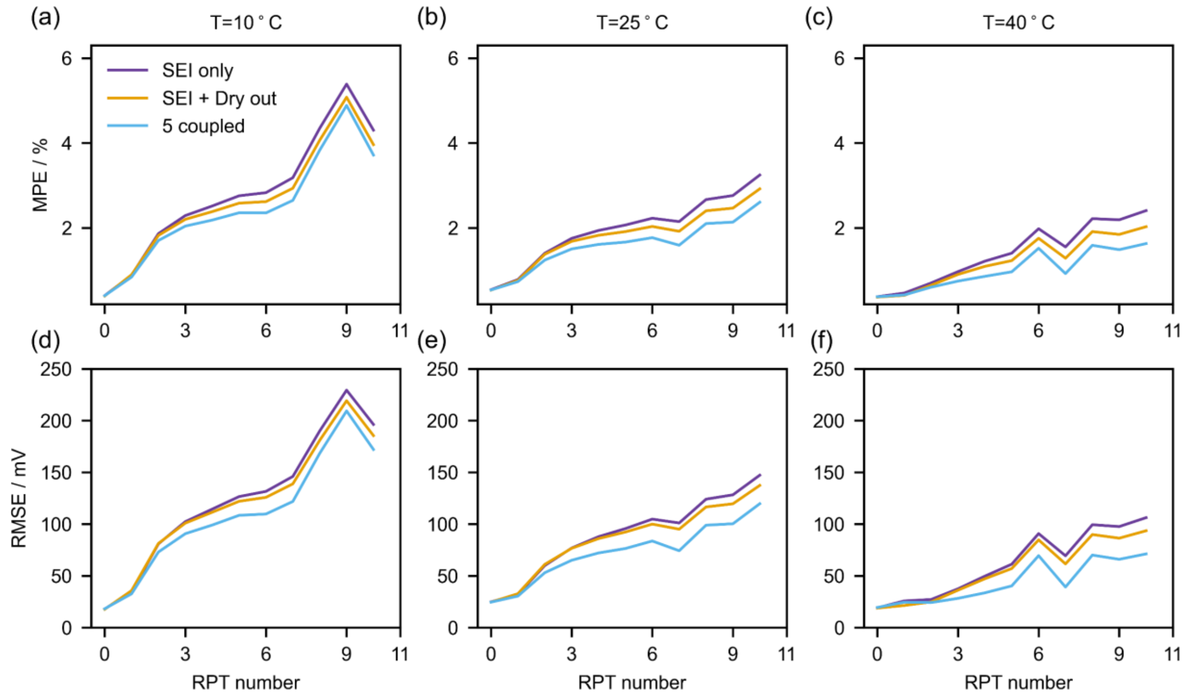

Supplementary Figure 15. MPE (a ~ c) and RMSE (d ~ f) of C/10 voltage during RPTs for Experiment 3 at three temperatures. Source data for this figure are provided as a Source Data file.

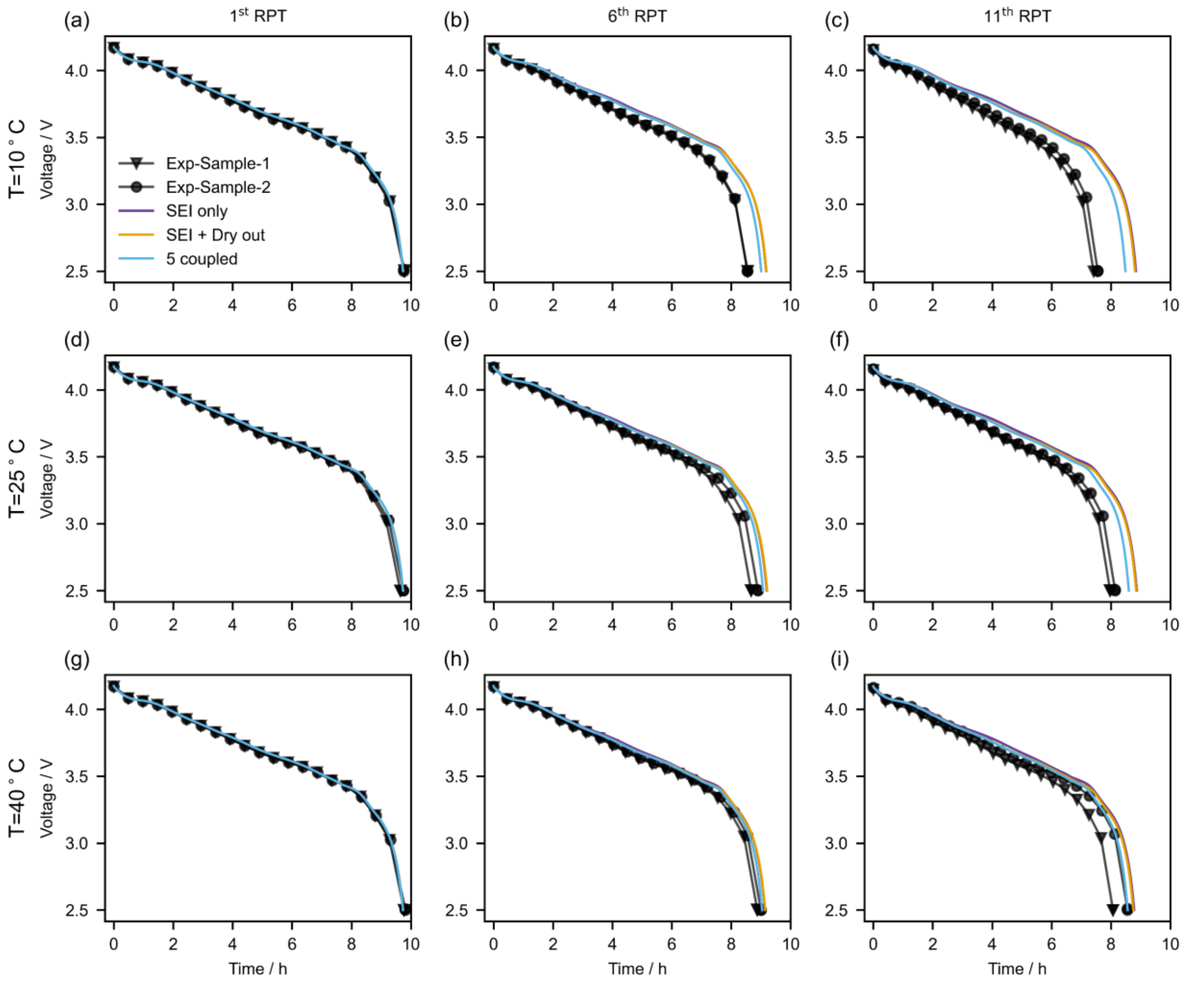

Supplementary Figure 16. C/10 discharge voltage at for cells aged at 10°C (a ~ c), 25°C (d ~ f), and 40°C (g ~ i) for Experiment 3. Note that all RPT are carried out at 25°C, the temperature just denotes their ageing temperature. Source data for this figure are provided as a Source Data file.

Supplementary Table 1. Mean RMSE of voltage curves for all C/10 discharge RPT cycles for Experiment 2.

Model	T / ℃	Mean RMSE / mV	Standard derivation of RMSE / mV
SEI only	10	34.91	5.35
SEI only	25	31.42	4.16
SEI only	40	39.39	4.42
SEI + Dry out	10	32.40	5.23
SEI + Dry out	25	29.26	8.51
SEI + Dry out	40	35.18	9.72
5 coupled	10	23.29	7.10
5 coupled	25	25.31	9.75
5 coupled	40	28.45	10.17

Supplementary Table 2. Equations of the DFN model[1].

Description	Equation	Boundary conditions
	**Electrodes**	
Mass conservation	$\frac{\partial c_k}{\partial t} = \frac{1}{r^2}\frac{\partial}{\partial r}\left(r^2 D_k \frac{\partial c_k}{\partial r}\right)$	$\left.\frac{\partial c_k}{\partial r}\right\rvert_{r=0} = 0, -D_k \left.\frac{\partial c_k}{\partial r}\right\rvert_{r=R_k} = \frac{j_k}{a_k F}$
Charge conservation	$\frac{\partial}{\partial x}\left(\sigma_k \frac{\partial \phi_k}{\partial x}\right) = j_k$	$-\sigma_\mathrm{n} \left.\frac{\partial \phi_\mathrm{n}}{\partial x}\right\rvert_{x=0} = -\sigma_\mathrm{p} \left.\frac{\partial \phi_\mathrm{p}}{\partial x}\right\rvert_{x=L} = i_\mathrm{app}$ $-\sigma_\mathrm{n} \left.\frac{\partial \phi_\mathrm{n}}{\partial x}\right\rvert_{x=L_\mathrm{n}} = -\sigma_\mathrm{p} \left.\frac{\partial \phi_\mathrm{p}}{\partial x}\right\rvert_{x=L-L_\mathrm{p}} = 0$
	**Electrolyte**	
Mass conservation	$\varepsilon_k \frac{\partial c_\mathrm{e}}{\partial t} = -\frac{\partial}{\partial x}\left(-\varepsilon_k^b D_\mathrm{e} \frac{\partial c_\mathrm{e}}{\partial x} + t_+^0 \frac{i_\mathrm{e}}{F}\right) + \frac{a_k j_k^\mathrm{tot}}{F}$	$\left.\frac{\partial c_\mathrm{e}}{\partial x}\right\rvert_{x=0} = \left.\frac{\partial c_\mathrm{e}}{\partial x}\right\rvert_{x=L} = 0$
Charge conservation	$\frac{\partial}{\partial x}\left(\varepsilon_k^b \sigma_\mathrm{e}\left(\frac{\partial \phi_\mathrm{e}}{\partial x} - \frac{2(1-t_0^+)R\,T}{F}\frac{\partial \ln c_\mathrm{e}}{\partial x}\right)\right) = -j_k$	$\left.\frac{\partial \phi_\mathrm{e}}{\partial x}\right\rvert_{x=0} = \left.\frac{\partial \phi_\mathrm{e}}{\partial x}\right\rvert_{x=L} = 0$
	**Reaction kinetics**	
Butler-Volmer	$j_k = \begin{cases} a_k\, j_{0,k}\, \sinh\left(\frac{RT}{2F}\eta_k\right), & k \in \{\mathrm{n,p}\}, \\ 0, & k = \mathrm{s}. \end{cases}$	
Exchange current	$j_{0,k} = k_k \sqrt{c_\mathrm{e} c_k (c_k^\mathrm{max} - c_k)}\big\rvert_{r=R_k}$	
Overpotential	$\eta_k = \phi_k - \phi_\mathrm{e} - U_k\left(c_k\rvert_{r=R_k}\right), \quad k \in \{\mathrm{n,p}\}$	
	**Initial conditions**	
Initial conditions	$c_k = c_{k0}, c_\mathrm{e} = c_{\mathrm{e}0}$	
	**Terminal voltage**	
Terminal voltage	$V = \phi_\mathrm{p}\big\rvert_{x=L} - \phi_\mathrm{n}\big\rvert_{x=0}$	

Supplementary Table 3. Thermal model considered in this work[1].

Description	Equation
Energy conservation	$\rho C_{\mathrm{p}}\frac{\partial T}{\partial t}=\nabla\cdot(k\nabla \mathrm{T})+q_{\mathrm{tot}}$
Total battery heat	$q_{\mathrm{tot}}=q_{\mathrm{rev}}+q_{\mathrm{j}}+q_{\mathrm{r}}$
Reversible heat, q_{rev}	$q_{\mathrm{rev}}=\frac{\int_0^L j_k T\left(\frac{\partial U}{\partial T}\right)dx}{L}$
Entropy change	$\Delta S=nF\frac{\partial U}{\partial T}$
Joule heat, q_{j}	$q_{\mathrm{j}}=\frac{\int_0^L\left[\sigma_{\mathrm{eff}}\left(\frac{\partial \phi_s}{\partial x}\right)^2+\kappa_{\mathrm{eff}}\left(\frac{\partial \phi_{\mathrm{e}}}{\partial x}\right)^2+\frac{2k_{\mathrm{eff}}RT}{F}(1-t_+^0)\frac{\partial(\ln c_{\mathrm{e}})}{\partial x}\frac{\partial \phi_{\mathrm{e}}}{\partial x}\right]dx}{l}$
Reaction heat, q_{r}	$q_{\mathrm{r}}=\frac{\int_0^L j_k(\phi_{\mathrm{s}}-\phi_{\mathrm{e}}-U)dx}{l}$
Convection boundary condition	$-k\frac{\partial T}{\partial n}=h(T-T_{\mathrm{amb}})$

Supplementary Table 4. Five different cases considered in the solvent consumption model.

Cases	Conditions	Volume exchange	Jelly roll and reservoir	R_{dry}	Species concentration
1	$dV_{\text{e}}^{\text{need}} > 0$ $V_{\text{e}}^{\text{res}}(t) \geq dV_{\text{e}}^{\text{need}}$	$dV_{\text{e}}^{\text{add}} = dV_{\text{e}}^{\text{need}}$ $dV_{\text{e}}^{\text{squeeze}} = 0$	$V_{\text{e}}(t+dt) = V_{\text{e}}(t) + dV_{\text{pore}}$ $V_{\text{e}}^{\text{res}}(t+dt) = V_{\text{e}}^{\text{res}}(t) - (dV_{\text{pore}} - dV_{\text{EC}})$	$R_{\text{dry}} = 1$	$c_{\text{e}}^{\text{res}}(t+dt) = c_{\text{e}}^{\text{res}}(t)$ $c_{\text{EC}}^{\text{res}}(t+dt) = c_{\text{EC}}^{\text{res}}(t)$ $c_{\text{e}}^{\text{avg}}(t+dt) = \frac{c_{\text{e}}^{\text{avg}}(t) \cdot V_{\text{e}} + c_{\text{e}}^{\text{res}}(t) \cdot \left(dV_{\text{pore}} - dV_{\text{EC}}\right)}{V_{\text{e}} + dV_{\text{pore}}}$ $c_{\text{EC}}(t+dt) = \frac{c_{\text{EC}}(t) \cdot (V_{\text{e}} + dV_{\text{EC}}) + c_{\text{EC}}^{\text{res}}(t) \cdot \left(dV_{\text{pore}} - dV_{\text{EC}}\right)}{V_{\text{e}} + dV_{\text{pore}}}$
2	$dV_{\text{e}}^{\text{need}} > 0$ $V_{\text{e}}^{\text{res}}(t) < dV_{\text{e}}^{\text{need}}$	$dV_{\text{e}}^{\text{add}} = V_{\text{e}}^{\text{res}}(t)$ $dV_{\text{e}}^{\text{squeeze}} = 0$	$V_{\text{e}}(t+dt) = V_{\text{e}}(t) + dV_{\text{EC}} + V_{\text{e}}^{\text{res}}$ $V_{\text{e}}^{\text{res}}(t+dt) = 0$	$R_{\text{dry}} < 1$	$c_{\text{e}}^{\text{res}}(t+dt) = 0$ $c_{\text{EC}}^{\text{res}}(t+dt) = 0$ $c_{\text{e}}^{\text{avg}}(t+dt) = \frac{c_{\text{e}}^{\text{avg}}(t) \cdot V_{\text{e}} + c_{\text{e}}^{\text{res}}(t) \cdot V_{\text{e}}^{\text{res}}(t)}{V_{\text{e}} + dV_{\text{EC}} + V_{\text{e}}^{\text{res}}(t)}$ $c_{\text{EC}}(t+dt) = \frac{c_{\text{EC}}(t) \cdot (V_{\text{e}} + dV_{\text{EC}}) + c_{\text{EC}}^{\text{res}}(t) \cdot V_{\text{e}}^{\text{res}}(t)}{V_{\text{e}} + dV_{\text{EC}} + V_{\text{e}}^{\text{res}}(t)}$
3	$dV_{\text{e}}^{\text{need}} = 0$	$dV_{\text{e}}^{\text{squeeze}} = 0$ $dV_{\text{e}}^{\text{add}} = 0$	$V_{\text{e}}(t+dt) = V_{\text{e}}(t) + dV_{\text{EC}}$ $V_{\text{e}}^{\text{res}}(t+dt) = V_{\text{e}}^{\text{res}}(t)$	$R_{\text{dry}} = 1$	$c_{\text{e}}^{\text{res}}(t+dt) = c_{\text{e}}^{\text{res}}(t)$ $c_{\text{EC}}^{\text{res}}(t+dt) = c_{\text{EC}}^{\text{res}}(t)$ $c_{\text{e}}^{\text{avg}}(t+dt) = c_{\text{e}}^{\text{avg}}(t)$ $c_{\text{EC}}(t+dt) = c_{\text{EC}}(t)$
4	$dV_{\text{e}}^{\text{need}} < 0$ $V_{\text{e}}^{\text{res}}(t) = 0$ $A_{\text{cell}}(t) < A_{\text{cell,max}}$	$dV_{\text{e}}^{\text{add}} = 0$ $dV_{\text{e}}^{\text{squeeze}} = 0$	$V_{\text{e}}(t+dt) = V_{\text{e}}(t) + dV_{\text{EC}}$ $V_{\text{e}}^{\text{res}}(t+dt) = 0$	$R_{\text{dry}} > 1$	$c_{\text{e}}^{\text{res}}(t+dt) = c_{\text{e}}^{\text{res}}(t)$ $c_{\text{EC}}^{\text{res}}(t+dt) = c_{\text{EC}}^{\text{res}}(t)$ $c_{\text{e}}^{\text{avg}}(t+dt) = c_{\text{e}}^{\text{avg}}(t)$ $c_{\text{EC}}(t+dt) = c_{\text{EC}}(t)$
5	$dV_{\text{e}}^{\text{need}} < 0$ $A_{\text{cell}} = A_{\text{cell,max}}$	$dV_{\text{e}}^{\text{add}} = 0$ $dV_{\text{e}}^{\text{squeeze}} = -dV_{\text{e}}^{\text{need}}$	$V_{\text{e}}(t+dt) = V_{\text{e}}(t) + dV_{\text{EC}} + dV_{\text{e}}^{\text{need}}$ $V_{\text{e}}^{\text{res}}(t+dt) = V_{\text{e}}^{\text{res}}(t) - dV_{\text{e}}^{\text{need}}$	$R_{\text{dry}} = 1$	$c_{\text{e}}^{\text{res}}(t+dt) = \frac{c_{\text{e}}^{\text{res}}(t) \cdot V_{\text{e}}^{\text{res}} - \left(dV_{\text{pore}} - dV_{\text{EC}}\right) \cdot c_{\text{e}}^{\text{avg}}}{V_{\text{e}}^{\text{res}} - dV_{\text{pore}} + dV_{\text{EC}}}$ $c_{\text{EC}}^{\text{res}}(t+dt) = \frac{c_{\text{EC}}^{\text{res}}(t) \cdot V_{\text{e}}^{\text{res}} - \left(dV_{\text{pore}} - dV_{\text{EC}}\right) \cdot c_{\text{EC}}}{V_{\text{e}}^{\text{res}} - dV_{\text{pore}} + dV_{\text{EC}}}$ $c_{\text{e}}^{\text{avg}}(t+dt) = c_{\text{e}}^{\text{avg}}(t)$ $c_{\text{EC}}(t+dt) = c_{\text{EC}}(t)$

Supplementary Table 5. Summary of the five experiments, showing conditions used and distribution of cells, adapted from [3].

Experiment	SOC window	Cycles per ageing set	Current (discharge/charge)	Temperature / ℃	Number of cells
1	0~30%	258	0.3C/1C	10	3
				25	3
				40	3
2	70~85%	515	0.3C/1C	10	2
				25	2
				40	2
3	85~100%	515	0.3C/1C	10	3
				25	3
				40	3
4	0~100% (drive cycle)	78	0.3C/ noisy C	10	3
				25	2
				40	3
5	0~100%	78	0.3C/1C	10	3
				25	2
				40	3

Supplementary Table 6. The parameters used for the DFN and thermal model in this study. All parameters are taken from O'Regan *et al.*[1] except for those marked with an asterisk (*), which have been changed for this work.

Type	Parameter	Unit	Positive electrode	Separator	Negative electrode
	Active material		$Li_xNi_{0.8}Mn_{0.1}Co_{0.1}O_2$	Ceramic coated polyolefin	Li_xC_6 + SiO_x
Design specifications	Current collector thickness (L_{CC})	M	1.6×10^{-5}		1.2×10^{-5}
	Current collector conductivity (σ_{CC})	S/m	3.6914×10^{7}		5.8411×10^{7}
	Current collector density (ρ_{CC})	kg/m^3	2700		8960
	Current collector specific heat capacity ($C_{p,cc}$)	J/kg/K	897		385
	Current collector thermal conductivity (λ_{cc})	W/m/K	237		401
	Electrode thickness (L)	M	7.56×10^{-5}	1.2×10^{-5}	8.52×10^{-5}
	Electrode length (w)	M		1.58	
	Electrode width (h)	M		6.5×10^{-2}	
	Cell cooling surface area (A_{cool})	m^2		5.31×10^{-3}	
	Cell volume (V_{cell})	m^3		2.42×10^{-5}	
	Cell thermal expansion coefficient (α_{th})	m/K		1.1×10^{-6}	
	Total heat transfer coefficient (h_{th})	W/m^2/K		20	
	Mean particle radius (R_s)	M	5.22×10^{-6}		5.86×10^{-6}
	Electrolyte volume fraction (ε_e)		0.335	0.47	See Eq. (S11)*
	Active material volume fraction (ε_s)		0.665		0.75
	Contact resistance (R_0)	mΩ		11.5	
	Bruggeman exponent (electrode) (b)		0	1.5	0
	Bruggeman exponent (electrolyte) (b)		1.5	1.5	1.5
Electrode	Solid phase lithium diffusivity (D_s)	m^2·s^{-1}	Eq. (S18)		Eq. (S18)
	Solid phase electronic conductivity (σ_s)	S·m^{-1}	Eq. (S19)		215

	Density (wet, $\rho_{\mathrm{s+l}}$)	$\mathrm{kg/m^3}$	3700	1548	2060
	Density (porous, ρ_{s})	$\mathrm{kg/m^3}$	3270	1740	946
	Poisson's ratio (ν)		0.2		0.3
	Young's modulus (E)	GPa	375		15
	Reference concentration for free of deformation (c_{ref})	$\mathrm{mol{\cdot}m^{-3}}$	0		0
	Partial molar volume (Ω)	$\mathrm{m^3/mol}$	1.25×10^{-5}		3.1×10^{-6}
	Wet electrode specific heat capacity ($\bar{\theta}$)	J/kg/K-1	Eq. (S20)		Eq. (S20)
	Thermal conductivity (wet, λ)	W/m/K	Eq. (S24)	0.3344	Eq. (S24)
	Maximum concentration ($c_{\mathrm{s}}^{\mathrm{max}}$)	$\mathrm{mol{\cdot}m^{-3}}$	52787*		32544*
	Initial concentration ($c_{\mathrm{s}}^{\mathrm{int}}$)	$\mathrm{mol{\cdot}m^{-3}}$	12727*		28543*
Electrolyte	Li^+ diffusivity in the electrolyte (D_{e})	$\mathrm{m^2{\cdot}s^{-1}}$		EC:EMC 3:7 wt% in $LiPF_6$, Eq. (S13)*	
	Electrolyte ionic conductivity (κ_{e})	$\mathrm{S{\cdot}m^{-1}}$		EC:EMC 3:7 wt% in $LiPF_6$, Eq. (S14) *	
	Cation transference number (t_+^0)	-		EC:EMC 3:7 wt% in $LiPF_6$, Eq. (S15) *	
	Thermodynamic factor (χ)	-		EC:EMC 3:7 wt% in $LiPF_6$, Eq. (S16) *	
	Initial Li^+ concentration in electrolyte in the jell roll ($c_{\mathrm{e,0}}$)	$\mathrm{mol{\cdot}m^{-3}}$		1000	
	Initial EC concentration in electrolyte in the jell roll ($c_{\mathrm{EC,0}}$)	$\mathrm{mol{\cdot}m^{-3}}$		4541	
	Heat capacity of electrolyte ($C_{\mathrm{p,l}}$)	$\mathrm{J/K/kg^3}$		229	
Intercalation reaction	OCPs (U_i^{OCP})	V	Eq. (S1), (S2)		Eq. (S3), (S4)*
	Entropy change ($\frac{\partial U_i}{\partial T}$)	V/K	Eq. (S5)		Eq. (S6)
	Exchange current density ($j_{0,k}^{\mathrm{int}}$)	$\mathrm{A/m^2}$	Eq. (S26)		Eq. (S27)

Supplementary Table 7. Fitting parameters for the function describing solid-phase electrode diffusivity[1]. A "-" means that the term including that parameter has not been included. The activation energy has been changed for this work.

Fitting Parameter	Positive Electrode	Negative Electrode
a_0	-	11.17
a_1	-0.9231	-1.553
a_2	-0.4066	-6.136
a_3	-0.993	-9.725
a_4	-	1.85
b_0	-13.96	-15.11
b_1	0.3216	0.2031
b_2	0.4532	0.5375
b_3	0.8098	0.9144
b_4	-	0.5953
c_1	0.002534	0.0006091
c_2	0.003926	0.06438
c_3	0.09924	0.0578
c_4	-	0.001356
E_{act}	12000	See Table 3 in Methods section in the main text*
R_{cor}	2.7	3.0321

Supplementary Table 8. Ageing parameters (related to the negative electrode only) that remain unchanged in this study.

Ageing mechanism	Parameter	Unit	Values
SEI	Ratio of lithium moles to SEI moles (z_{SEI})		2
	Lithium interstitial reference concentration ($c_{\mathrm{int,Li}}$)	mol/m^3	15
	SEI resistivity (ρ_{SEI})	$\Omega \cdot m$	2×10^5
	Inner SEI reaction proportion (α)	-	0.5
	Initial inner SEI thickness ($L_{\mathrm{SEI,inner,0}}$)	m	1.23625×10^{-8}
	Initial outer SEI thickness ($L_{\mathrm{SEI,outer,0}}$)	m	1.23625×10^{-8}
	Initial Li$^+$ concentration in electrolyte in the reservoir ($c_{\mathrm{Li^+,0}}^{\mathrm{res}}$)	mol/m^3	1000
	Initial EC concentration in electrolyte in the reservoir ($c_{\mathrm{EC,0}}^{\mathrm{res}}$)	mol/m^3	4541
Solvent consumption	Initial Li$^+$ concentration in electrolyte in the reservoir ($c_{\mathrm{Li^+,0}}^{\mathrm{res}}$)	mol/m^3	1000
	Initial excessive electrolyte amount	-	1.0
	Initial EC concentration in electrolyte in the reservoir ($c_{\mathrm{EC,0}}^{\mathrm{res}}$)	mol/m^3	4541
	EC partial molar volume ($\bar{V}_{\mathrm{EC}}$)	m^3/mol	6.667×10^{-5}
Lithium plating	Lithium plating transfer coefficient ($\alpha_{\mathrm{a,Li}}$)	-	0.65
	Initial plated lithium concentration ($c_{\mathrm{Li,0}}$)	mol/m^3	0
	Lithium metal partial molar volume ($\bar{V}_{\mathrm{Li}}$)	m^3/mol	1.3×10^{-5}

Supplementary Table 9. Ageing parameters (related to both electrodes) that remain unchanged in this study.

Ageing mechanism	Parameter	Unit	Positive	Negative
LAM model	LAM exponential term (m_2)	-	2	2
Mechanical and cracking	Electrode stress intensity factor correction (b_{cr})	-	1.12	1.12
	Paris' law exponential term (m_{cr})		2.2	2.2
	Number of cracks per unit area ($\rho_{\mathrm{cr}}^{\mathrm{neg}}$)	1/m^2	3.18×10^{15}	3.18×10^{15}
	Initial crack length ($l_{\mathrm{cr,0}}^{\mathrm{pos}}$)	m	2×10^{-8}	2×10^{-8}
	Initial crack width ($w_{\mathrm{cr,0}}^{\mathrm{pos}}$)	m	1.5×10^{-8}	1.5×10^{-8}
	Electrode critical stress (σ_{c})	MPa	375	60

Supplementary Table 10. Average values and standard derivations of RMSEs of the C/10 voltage curves for all RPT cycles in Experiment 3.

Model	T / ℃	Mean RMSE / mV	Standard derivation of RMSE / mV
SEI only	10	124.58	62.42
SEI only	25	89.35	36.86
SEI only	40	62.17	31.09
SEI + Dry out	10	119.96	58.57
SEI + Dry out	25	85.64	33.49
SEI + Dry out	40	56.49	27.59
5 coupled	10	109.42	55.22
5 coupled	25	72.58	27.54
5 coupled	40	44.20	19.81

Supplementary Table 11. Average values and standard derivations of MPEs of the C/10 voltage curves for all RPT cycles in Experiment 3.

Model	T / ℃	Mean MPE / %	Standard derivation of MPE / %
SEI only	10	2.80	1.42
SEI only	25	1.96	0.78
SEI only	40	1.41	0.69
SEI + Dry out	10	2.63	1.31
SEI + Dry out	25	1.80	0.68
SEI + Dry out	40	1.22	0.58
5 coupled	10	2.45	1.25
5 coupled	25	1.59	0.57
5 coupled	40	1.01	0.45

Supplementary Table 12. Mean percentage error for all degradation modes, models, and temperatures. The $\mathrm{MPE_{tot}}$ column is a weighted index defined in Eq. (1) for Experiment 3.

Model	T / °C	$\mathrm{MPE_{SOH}}$ / %	$\mathrm{MPE_{Res}}$ / %	$\mathrm{MPE_{LLI}}$ / %	$\mathrm{MPE_{LAM_{NE}}}$ / %	$\mathrm{MPE_{LAM_{PE}}}$ / %	$\mathrm{MPE_{tot}}$ / %
SEI only	10	7.14	7.36	54.8	100	100	36.3
SEI only	10	7.19	6.22	48.9	83.06	67.03	29.2
SEI only	10	5.3	6.95	38.1	81.06	61.45	26.1
SEI + Dry out	25	4.02	3.14	49.51	100.00	100.00	33.59
SEI + Dry out	25	4.06	1.85	42.38	53.44	60.34	21.78
SEI + Dry out	25	2.44	2.64	28.67	32.63	54.58	16.04
5 coupled	40	1.98	6.07	42.58	100.00	100.00	32.07
5 coupled	40	1.97	4.22	33.08	40.24	38.39	15.47
5 coupled	40	0.75	5.86	22.67	19.82	33.32	10.58

Supplementary Note 1

Experiment details

The commercial 21700 cylindrical cells (LGM50T, LG GBM50T2170) were cycled between 70%~85%SOC under 10, 25 and 40 ℃. It has a SiO_x-doped graphite electrode alongside an NMC811 positive electrode, with a nominal 1C capacity of 5 Ah.

The overall test procedure includes a break-in test at the beginning of life (BOL), followed by repeated reference performance tests (RPT) and ageing tests. The break-in test is designed to bring different samples of a batch of cells into the same and stable conditions before the degradation study. It includes five full discharge−charge cycles at a rate of 0.2C. Two RPT tests are performed after the break-in test, namely a long one (last ~100 hours) and a short one (last ~50 hours), followed by an ageing test. After the ageing tests, the long RPT is performed after those even number of ageing tests, whereas the short RPT is performed after those odd number of ageing tests. The long RPT test contains 4 subsets, namely a full charge-discharge cycle at (i) 0.1C and (ii) 0.5C, and two galvanostatic intermittent titration technique (GITT) discharge tests at 0.5C with (iii) 25 and (iv) 5 pulses. For the 25-pulse GITT subset, 200 mAh of charge is passed, followed by a rest period of one hour. The 5-pulse GITT subset will pass 1000 mAh of charge, separated by the same rest period. To avoid overcharge/over-discharge, the lower and upper voltage limit for the full charge discharge cycle during the whole experiment are 2.5 V and 4.2 V, respectively. Note that in order to shorten the simulation time on RPTs, we have only model the three processes of the long RPT: (i) 0.1C and (ii) 0.5C, and (iii) one GITT at 0.5C with 25 pulses. in order to maintain the same charge throughput in RPTs, we have repeated the 0.5C constant-current cycling twice.

For the ageing test, five experiments are carried out, with detailed conditions listed in Supplementary Table 5. During these experiments (including RPTs and ageing tests), all the cells are fixed in bespoke test rigs which maintain a constant temperature on the base of the cells, with pseudo-adiabatic temperature conditions on the other surfaces.

The data extracted from the experiment for model validation in this paper are mainly from the RPT test, which includes the 0.1C discharge capacities, the 0.1C discharge voltage curves, the 0.1s resistance and the degradation modes (DMs) extracted from the 0.1C discharge voltage curves. Specifically, the 0.1s resistance is extracted from the 25-pulse GITT data based on the instantaneous potential drop upon applying the current pulse:

$$\mathrm{R}_{0.1\mathrm{s}} = \frac{V_2 - V_1}{I_2 - I_1} \quad \text{(S1)}$$

Among which V_1 and I_1 are the voltage and current during rest before applying the current pulse, and V_2 and I_2 are the values immediately after the current pulse. The term "immediately" here refers to 0.1 second here because the sampling rate of the GITT test is set to 10 Hz. Within such short period, the main contribution of the resistance should originate from the ohmic part (contact resistance, electrolyte conductivity, solid conductivity, etc.) and the charge transfer part, whereas the polarization part can be ignored. Note that using the above equations we can get 25 values of 0.1 resistance at different SOC. To compare the resistance changing over time and make it easier for model validation, we have picked the resistance at the 12th pulse, roughly corresponding to 52% SOC of the cells at BOT. This is because the resistance is relatively flat in this region.

Supplementary Note 2

SEI growth model

O'Kane *et al.*[4] chose a simple solvent-diffusion limited model of SEI growth, on the grounds that it was able to predict the square root of time dependence observed throughout the literature, with only one fitting parameter. However, the solvent-diffusion limited model has no dependence on SoC, despite SoC dependence also being observed throughout the literature [5-7]. In this work, we follow the assumption made by Single *et al.*[8] and von Kolzenberg *et al.*[9] that the SEI reaction rate is limited by the diffusion of neutral lithium interstitials within the inner SEI layer, leading to an expression for the interfacial SEI current density $[\mathrm{A/m^2}]$:

$$j_+^{\mathrm{SEI}} = \frac{c_{\mathrm{int,Li}}}{L_{\mathrm{SEI}}^{\mathrm{inner}}} \cdot D_{\mathrm{int}} F \cdot \exp\left(-\phi_{\mathrm{s}} + \phi_{\mathrm{e}}\right), \tag{S2}$$

where $L_{\mathrm{SEI}}^{\mathrm{inner}}$ $[\mathrm{m}]$ is the thickness of the inner SEI layer. c_{int} $[\mathrm{mol/m^3}]$ and D_{int} $[\mathrm{m^2/s}]$ are the concentration and diffusivity of the neutral lithium interstitials in the SEI layer, respectively, which are assumed to be the same for the inner and outer SEI layers.

Due to the lack of information on the growth rate or current distribution between the inner and outer layer, we assume these two layers grow at the same rate:

$$j_+^{\mathrm{SEI,inner}} = 0.5 j_+^{\mathrm{SEI}}, \tag{S3}$$

$$j_+^{\mathrm{SEI,outer}} = 0.5 j_+^{\mathrm{SEI}}, \tag{S4}$$

The temperature-dependent SEI current density is:

$$j_+^{\mathrm{SEI}}(T) = j_+^{\mathrm{SEI}}(T_{\mathrm{ref}}) \cdot \exp\left(\frac{E_{\mathrm{SEI}}}{RT_{\mathrm{ref}}} - \frac{E_{\mathrm{SEI}}}{RT}\right), \tag{S5}$$

where E_{SEI} $[\mathrm{J/mol}]$ is the activation energy of the SEI reaction, and T_{ref} $[\mathrm{K}]$ is the reference temperature, set to 298.15 K (25°C) in this study.

The SEI thickness increases as follows:

$$\frac{dL_{\mathrm{SEI,inner}}}{dt} = \frac{j_+^{\mathrm{SEI,inner}}}{2F} \cdot \bar{V}_{\mathrm{SEI}}^{\mathrm{inner}} = \frac{c_{\mathrm{int}}}{2L_{\mathrm{SEI}}^{\mathrm{inner}}} \cdot D_{\mathrm{int}} \bar{V}_{\mathrm{SEI}}^{\mathrm{inner}} \cdot e^{-(\phi_{\mathrm{s}} - \phi_{\mathrm{e}})} \tag{S6}$$

$$\frac{dL_{\mathrm{SEI,outer}}}{dt} = \frac{j_{+}^{\mathrm{SEI,outer}}}{2F} \cdot \bar{V}_{\mathrm{SEI}}^{\mathrm{outer}} = \frac{c_{\mathrm{int}}}{2L_{\mathrm{SEI}}^{\mathrm{outer}}} \cdot D_{\mathrm{int}} \bar{V}_{\mathrm{SEI}}^{\mathrm{outer}} \cdot e^{-(\phi_{\mathrm{s}} - \phi_{\mathrm{e}})} \tag{S7}$$

where F [C/mol] and a_n [1/m] are the Faraday constant and the specific surface area, respectively. For spherical particles $a_n = 3\varepsilon_n / R_n$, where R_n [m] is the radius of the negative electrode particles. $\bar{V}_{\mathrm{SEI}}^{\mathrm{inner}}$ and $\bar{V}_{\mathrm{SEI}}^{\mathrm{outer}}$ [m^3/mol] are the partial molar volumes of the inner and outer SEI layer, respectively.

The total SEI thickness is the summation of the inner and outer SEI thicknesses:

$$L_{\mathrm{SEI}} = L_{\mathrm{SEI,inner}} + L_{\mathrm{SEI,outer}} \tag{S8}$$

The SEI has an Ohmic resistivity ρ_{SEI} $[\Omega \cdot \mathrm{m}]$, which results in an overpotential η_{SEI} [V]:

$$\eta_{\mathrm{SEI}} = \rho_{\mathrm{SEI}} \cdot j_n^{\mathrm{tot}} \cdot L_{\mathrm{SEI}}, \tag{S9}$$

where j_n^{tot} [A/m^2] is the total interfacial current density in the negative electrode.

This interstitial-diffusion limited model is an improvement on the solvent-diffusion limited model chosen, because it captures both time dependence and SoC dependence. While it contains two adjustable parameters, c_{int} and D_{int}, these act as one parameter because they appear in Eqs. (6) and (7) as a product of each other, but never on their own.

Supplementary Note 3

Lithium plating model

The lithium plating model in this study is unchanged from O'Kane *et al.*[4], who used a partially reversible plating model in which plating, stripping and dead lithium formation occur at the same time. The plating and stripping reactions are governed by a Butler-Volmer equation:

$$j_{\mathrm{Li}} = Fk_{\mathrm{Li}}\left(c_{\mathrm{Li}}\exp\left(\frac{F\alpha_{\mathrm{a,Li}}(\phi_{\mathrm{s}}-\phi_{\mathrm{e}}-\eta_{\mathrm{SEI}})}{RT}\right) - c_{\mathrm{e}}\exp\left(-\frac{F\alpha_{\mathrm{c,Li}}(\phi_{\mathrm{s}}-\phi_{\mathrm{e}}-\eta_{\mathrm{SEI}})}{RT}\right)\right), \quad \text{(S10)}$$

where j_{Li} [A/m^2] is the lithium plating interfacial current density, k_{Li} [m/s] is the lithium plating kinetic rate constant,

$$\frac{\partial c_{\mathrm{Li}}}{\partial t} = -\frac{a_n j_{\mathrm{Li}}}{F} - \frac{\partial c_{\mathrm{dl}}}{\partial t}, \quad \text{(S11)}$$

where c_{dl} [mol/m^3] is the concentration of the dead lithium, with time evolution given by:

$$\frac{\partial c_{\mathrm{dl}}}{\partial t} = \gamma c_{\mathrm{Li}}. \quad \text{(S12)}$$

γ [1/s] is a decay rate, defined as:

$$\gamma = \gamma_0 \cdot \frac{L_{\mathrm{SEI,0}}}{L_{\mathrm{SEI}}}, \quad \text{(S13)}$$

where γ_0 [1/s] is the decay rate constant, a fitting parameter, and $L_{\mathrm{SEI,0}}$ is the initial thickness of the SEI layer. The dependence of the decay rate on L_{SEI} is designed to account for the role played by the solvent molecules in the electrolyte in the transition from plated lithium to dead lithium.

Supplementary Note 4

Electrode particle cracking model

The particle cracking is induced by cyclic stress. Therefore, we need to introduce the classic stress model first. We implement the stress model originally proposed by Zhang et al.[10], based on the equilibrium of stresses for a free-standing spherical electrode particle. The analytical solutions for the radial stress σ_{r}, tangential stress σ_{t} and displacement u are:

$$\sigma_{\mathrm{r}} = \frac{2\Omega E}{(1-\upsilon)} \cdot [c_{\mathrm{avg}}(R_i) - c_{\mathrm{avg}}(r)], \tag{S14}$$

$$\sigma_{\mathrm{t}} = \frac{\Omega E}{(1-\upsilon)} \cdot [2c_{\mathrm{avg}}(R_i) + c_{\mathrm{avg}}(r) - \bar{c}/3], \tag{S15}$$

$$u = \frac{(1+\upsilon)}{(1-\upsilon)} \cdot \Omega r c_{\mathrm{avg}}(r) + \frac{2(1-2\upsilon)}{(1-\upsilon)} \Omega r c_{\mathrm{avg}}(R_i), \tag{S16}$$

where Ω is the partial molar volume [m^3/mol], E is the Young's modulus [Pa], υ is the Possion's ratio, R_i [m] is the particle radius and $c_{\mathrm{avg}}(r)$ [mol/m^3] is the average Li$^+$ concentration between 0 and r:

$$c_{\mathrm{avg}}(r) = \frac{1}{3r^3} \int_0^r \bar{c} r^2 \mathrm{d}r, \tag{S17}$$

where $\bar{c} = c - c_{\mathrm{ref}}$ is the deviation in lithium concentration from the reference value c_{ref} for the stress-free case.

Deshpande et al.[11] assumes that the tensile tangential stress ($\sigma_{\mathrm{t}} > 0$ [Pa]) induces identical micro cracks on the electrode particle surface. Three parameters are proposed to describe these cracks, namely the length l_{cr} [m], width w_{cr} [m], and density (number of cracks per unit electrode surface area) ρ_{cr} [1/m^2]. It is further assumed that these cracks grow in length during cycling, but maintain the same width and density. The growth of the crack length follows Paris' law:

$$\frac{dl_{\mathrm{cr}}}{dt} = \frac{1}{t_0} \cdot \frac{dl_{\mathrm{cr}}}{dN} = \frac{k_{\mathrm{cr}}}{t_0} \left(\sigma_{\mathrm{t}} b_{\mathrm{cr}} \sqrt{\pi l_{\mathrm{cr}}}\right)^{m_{\mathrm{cr}}} \text{ for } \sigma_{\mathrm{t}} > 0, \tag{S18}$$

where t_0 [s] is the time for one cycle, b_{cr} is the stress intensity factor correction, k_{cr} and m_{cr} are constants that are determined from experimental data. The instantaneous rate of change of the crack area to volume ratio [1/m] can be estimated by:

$$\frac{da_{\mathrm{cr}}}{dt} = \frac{a_{\pm}\rho_{\mathrm{cr}} w_{\mathrm{cr}}}{t_0} \cdot \frac{dl_{\mathrm{cr}}}{dt} = \frac{a_{\pm}\rho_{\mathrm{cr}} w_{\mathrm{cr}}}{t_0} \cdot k_{\mathrm{cr}} \left(\sigma_{\mathrm{t}} b_{\mathrm{cr}} \sqrt{\pi l_{\mathrm{cr}}}\right)^{m_{\mathrm{cr}}} \text{ for } \sigma_{\mathrm{t}} > 0. \tag{S19}$$

For interactions between the SEI growth and particle cracking, we can apply the same SEI growth model on the cracks. However, the SEI formation on the newly exposed fresh crack surfaces is expected to be faster than on those surfaces with existing SEI layers. As a result, the SEI layer thickness is not uniform along cracks, because crack propagation leads to different exposure times for different interface locations along a crack. To avoid having different SEI thickness along cracks and the mathematical and numerical complications this would create, we have used a recalculated, averaged thickness of the SEI layer on cracks as the fundamental measure of SEI-on-cracks sub-model, which is governed by:

$$\frac{\partial L_{\mathrm{SEI,cr}}}{\partial t} = \frac{c_{\mathrm{int,Li}}}{2L_{\mathrm{SEI,cr}}} \cdot D_{\mathrm{int}} \bar{V}_{\mathrm{SEI}} \cdot e^{-(\phi_{\mathrm{s}} - \phi_{\mathrm{e}})} - \frac{\partial l_{\mathrm{cr}}}{\partial t} \cdot \frac{L_{\mathrm{SEI,cr}}}{l_{\mathrm{cr}}}. \quad \text{(S20)}$$

In the above equation, $L_{\mathrm{SEI,cr}}$ [m] time evolution has two contributions: (1) the existing SEI layers are growing (first term on the right), and (2) the cracks expose fresh surfaces, which reduce the average SEI thickness on cracks (second term on the right).

Supplementary Note 5

Mechanical loss of active material model

We include the effect of loss of active material (LAM) due to particle cracking in either electrode. The following key equation is taken from Laresgoiti *et al.*[12] and Reniers *et al.*[13], and simplified by O'Kane *et al.*[4]:

$$\frac{\partial \varepsilon_{\mathrm{a}}}{\partial t} = \frac{\beta}{t_0} \cdot \left(\frac{\sigma_{\mathrm{h}}}{\sigma_{\mathrm{c}}}\right)^{m_2} \text{ for } \sigma_{\mathrm{h}} > 0, \tag{S21}$$

where ε_{a} is the volume fraction of active materials, β and m_2 are the LAM proportional and exponential terms, respectively, σ_{c} [Pa] is the critical stress of the electrode materials, and σ_{h} [Pa] is the hydrostatic stress, a function of radial (σ_{r}) and tangential (σ_{t}) stress:

$$\sigma_{\mathrm{h}} = (\sigma_{\mathrm{r}} + 2\sigma_{\mathrm{t}})/3. \tag{S22}$$

Supplementary Note 6

Solvent consumption model

The solvent consumption model is taken from Li *et al.* [14], which includes the presence of an electrolyte reservoir, outside of the jelly roll, but within the cell casing. To increase computational efficiency, Li *et al.*[14] accounted for solvent consumption not by introducing additional differential equations for the time evolution, but instead creating a coding wrapper around the main PyBaMM model. This wrapper calculates the amount of solvent consumed over a time interval Δt, then applies the effect of solvent consumption to the variables in the DFN model. The solvent consumption model in the present work is an improvement of the model proposed by Li *et al.*[14]. as the improvements consider the electrolyte being consumed by those SEI on cracks, and the possibility of electrolyte being squeezed out due to plated lithium. Therefore, it is necessary to introduce this model in detail here.

To start with, the solvent consumption model[14] has the following key assumptions:

1. Fresh LIBs contain extra electrolyte inside the cell package that resides outside the jelly roll. This extra electrolyte is called electrolyte reservoir.
2. Solute-volume effect is ignored. The solvent retains its initial volume from before having been mixed with the composite electrolyte. Li^+ does not contribute to the volume of the electrolyte due to its low partial molar volume [15].
3. Only EC is consumed during cell ageing, following this reaction [16-18]:

$$2\mathrm{Li}^+ + 2(\mathrm{C_3H_4O_3})(\mathrm{EC}) + 2e^- \leftrightarrow (\mathrm{CH_2OCO_2Li})_2 + \mathrm{C_2H_4}, \qquad \text{(S23)}$$

4. The time needed for mass transport and electrolyte mixing between the solvent in the reservoir and that in the jelly roll is negligible.
5. Inside the jelly roll, EC is homogenous across the electrode, i.e., the EC concentration is independent of location. This and the previous assumption are built upon the fact that the degradation mechanisms that consumes EC are long-term processes compared to EC transport, allowing EC to equilibrate inside the jelly roll.

To describe the electrolyte reservoir, three variables are needed: the volume of the reservoir $V_{\mathrm{e}}^{\mathrm{res}}(t)$, and the Li⁺ and EC concentrations in the reservoir, $c_{\mathrm{Li^+}}^{\mathrm{res}}(t)$ and $c_{\mathrm{EC}}^{\mathrm{res}}(t)$. Correspondingly, we use the electrode area $A_{\mathrm{cell}}(t)$, and the two concentrations $c_{\mathrm{e}}(t)$ and $c_{\mathrm{EC}}(t)$ to describe the electrolyte in the jelly roll. Note that if without superscript of "res", the species concentration always refers to those in the jelly roll.

The porosity reduction due to SEI growth on the surface of the negative particles as well as on cracks, as well as due to lithium plating (here both reversible and irreversible) is:

$$\frac{d\varepsilon_{\mathrm{n}}}{dt} = -\frac{d(L_{\mathrm{total}} \cdot a_{\mathrm{n}})}{dt}, \tag{S24}$$

where L_{total} [m] is the total thickness of the deposit, including SEI and plated lithium:

$$L_{\mathrm{total}} = L_{\mathrm{SEI}} + 2l_{\mathrm{cr}} w_{\mathrm{cr}} \rho_{\mathrm{cr}} L_{\mathrm{SEI,cr}} + c_{\mathrm{Li}} \cdot \frac{\bar{V}_{\mathrm{Li}}}{a_{\mathrm{n}}} + c_{\mathrm{dl}} \cdot \frac{\bar{V}_{\mathrm{Li}}}{a_{\mathrm{n}}}. \tag{S25}$$

Note that in Li *et al.*[14], only the contribution from SEI layer growth on the particle surface is considered (L_{SEI}), whereas in the above equation, the contributions from SEI on cracks, active lithium and dead lithium are included. For simplicity, we assume the partial molar volume of dead lithium is the same as that of active lithium, both expressed as $\bar{V}_{\mathrm{Li}}$. Assuming that pore volume change can only occur in the negative electrode, the total pore volume change of the cell [m³] will be:

$$dV_{\mathrm{pore}} = d\int_0^{L_{\mathrm{n}}} \varepsilon_n \cdot A_{\mathrm{cell}} dx, \tag{S26}$$

The solvent is consumed due to SEI growth (both on negative particle surfaces and negative particle cracks). Based on the SEI reaction in Eq. (S23), the ratio of Li⁺, solvent (assumed to be EC here), and SEI is 2:2:1. Therefore, the consumed EC quantity in mole due to SEI reaction is:

$$dn_{\mathrm{EC}} = 2dn_{\mathrm{SEI}} = -2 \cdot d\int_0^{L_{\mathrm{n}}} \left(L_{\mathrm{SEI}} + 2l_{\mathrm{cr,n}} w_{\mathrm{cr,n}} \rho_{\mathrm{cr,n}} L_{\mathrm{SEI,cr}}\right)/\bar{V}_{\mathrm{SEI}} \cdot a_n \cdot A_{\mathrm{cell}} dx, \tag{S27}$$

If the solute-volume effect is ignored, the EC volume [m³] consumed due to the SEI reaction is:

$$dV_{\mathrm{EC}} = dn_{\mathrm{EC}} \cdot \bar{V}_{\mathrm{EC}} = -2\bar{V}_{\mathrm{EC}} \cdot d\int_0^{L_{\mathrm{n}}} \left(L_{\mathrm{SEI}} + 2l_{\mathrm{cr,n}} w_{\mathrm{cr,n}} \rho_{\mathrm{cr,n}} L_{\mathrm{SEI,cr}}\right)/\bar{V}_{\mathrm{SEI}} \cdot a_n \cdot A_{\mathrm{cell}} dx, \tag{S28}$$

To simplify the expression, we define a new variable B_{SEIcr}:

$$B_{\mathrm{SEIcr}}(L_{\mathrm{SEI}}, L_{\mathrm{SEI,cr}}) = 2\bar{V}_{\mathrm{EC}} \cdot (L_{\mathrm{SEI}} + 2l_{\mathrm{cr,n}} w_{\mathrm{cr,n}} \rho_{\mathrm{cr,n}} L_{\mathrm{SEI,cr}})/\bar{V}_{\mathrm{SEI}}, \tag{S29}$$

Then Eq. (S28) becomes:

$$dV_{\mathrm{EC}} = -d\int_0^{L_\mathrm{n}} B_{\mathrm{SEIcr}} \cdot a_n \cdot A_{\mathrm{cell}} dx, \tag{S30}$$

Remember in this work, $dV_{\mathrm{pore}} < 0$ and $dV_{\mathrm{EC}} < 0$. As a combined result of EC consumption (dV_{EC}) and porosity change (dV_{pore}), the electrolyte volume inside jelly roll may become more or less than the pore volume, causing electrolyte exchange due to the hydraulic effect. To consider difference cases, we define the difference between dV_{EC} and dV_{pore} to be:

$$dV_{\mathrm{e}}^{\mathrm{need}} = dV_{\mathrm{pore}} - dV_{\mathrm{EC}} = d\left(\int_0^{L_\mathrm{n}} \varepsilon_n \cdot A_{\mathrm{cell}} dx + d\int_0^{L_\mathrm{n}} B_{\mathrm{SEIcr}} \cdot a_n \cdot A_{\mathrm{cell}} dx\right) \tag{S31}$$

However, the amount of solvent in the reservoir may not be sufficient to satisfy this requirement. We define $dV_{\mathrm{e}}^{\mathrm{add}}$ [m^3] as the electrolyte volume that actually being absorbed from the reservoir to the jelly roll region. In another case where the electrolyte volume inside jelly roll exceeds that of the pore, we define $dV_{\mathrm{e}}^{\mathrm{squeeze}}$ [m^3] to depict the electrolyte volume that transfers from jelly roll to reservoir. Overall, the electrolyte volume changes in the jelly roll and reservoir regions are:

$$dV_{\mathrm{e}} = dV_{\mathrm{EC}} + dV_{\mathrm{e}}^{\mathrm{add}} - dV_{\mathrm{e}}^{\mathrm{squeeze}} \tag{S32}$$

$$dV_{\mathrm{e}}^{\mathrm{res}} = -dV_{\mathrm{e}}^{\mathrm{add}} + dV_{\mathrm{e}}^{\mathrm{squeeze}} \tag{S33}$$

It is assumed that at time t, the volume of pore and electrolyte inside the jelly roll always equals:

$$V_{\mathrm{e}}(t) = V_{\mathrm{pore}}(t) \tag{S34}$$

The volume in the jelly roll and reservoir regions at time $t + dt$ are:

$$V_{\mathrm{e}}(t + dt) = V_{\mathrm{e}}(t) + dV_{\mathrm{e}} \tag{S35}$$

$$V_{\mathrm{e}}^{\mathrm{res}}(t + dt) = V_{\mathrm{e}}^{\mathrm{res}}(t) + dV_{\mathrm{e}}^{\mathrm{res}} \tag{S36}$$

Recall that to describe the solvent consumption model, six variables are to be tracked: $V_{\mathrm{e}}^{\mathrm{res}}(t)$, $c_{\mathrm{e}}^{\mathrm{res}}(t)$, $c_{\mathrm{EC}}^{\mathrm{res}}(t)$, $A_{\mathrm{cell}}(t)$, $c_{\mathrm{e}}(x, t)$ and $c_{\mathrm{EC}}(t)$. $V_{\mathrm{e}}^{\mathrm{res}}(t)$ is tracked by Eq. (S36). To track $A_{\mathrm{cell}}(t)$, we define a dry-out ratio:

$$R_{\mathrm{dry}} = \frac{V_{\mathrm{e}}(t+dt)}{V_{\mathrm{pore}}(t+dt)} = \frac{V_{\mathrm{e}}(t)+dV_{\mathrm{e}}}{V_{\mathrm{e}}(t)+dV_{\mathrm{pore}}} \tag{S37}$$

$$A_{\mathrm{cell}}(t+dt) = R_{\mathrm{dry}} \cdot A_{\mathrm{cell}}(t) \tag{S38}$$

The time evolution of the concentrations of Li ions and EC in the reservoir and jelly roll domains can be defined by:

$$c_{\mathrm{e}}^{\mathrm{res}}(t+dt) = \frac{n_{\mathrm{e}}^{\mathrm{res}}+dn_{\mathrm{e}}^{\mathrm{res}}}{V_{\mathrm{e}}^{\mathrm{res}}+dV_{\mathrm{e}}^{\mathrm{res}}} = \frac{c_{\mathrm{e}}^{\mathrm{res}}(t)\cdot(V_{\mathrm{e}}^{\mathrm{res}}-dV_{\mathrm{e}}^{\mathrm{add}})+dV_{\mathrm{e}}^{\mathrm{squeeze}}\cdot c_{\mathrm{e}}^{\mathrm{avg}}}{V_{\mathrm{e}}^{\mathrm{res}}-dV_{\mathrm{e}}^{\mathrm{add}}+dV_{\mathrm{e}}^{\mathrm{squeeze}}}, \tag{S39}$$

$$c_{\mathrm{EC}}^{\mathrm{res}}(t+dt) = \frac{n_{\mathrm{EC}}^{\mathrm{res}}+dn_{\mathrm{EC}}^{\mathrm{res}}}{V_{\mathrm{e}}^{\mathrm{res}}+dV_{\mathrm{e}}^{\mathrm{res}}} = \frac{c_{\mathrm{EC}}^{\mathrm{res}}(t)\cdot(V_{\mathrm{e}}^{\mathrm{res}}-dV_{\mathrm{e}}^{\mathrm{add}})+dV_{\mathrm{e}}^{\mathrm{squeeze}}\cdot c_{\mathrm{EC}}}{V_{\mathrm{e}}^{\mathrm{res}}-dV_{\mathrm{e}}^{\mathrm{add}}+dV_{\mathrm{e}}^{\mathrm{squeeze}}}, \tag{S40}$$

$$c_{\mathrm{e}}^{\mathrm{avg}}(t+dt) = \frac{n_{\mathrm{e}}+dn_{\mathrm{e}}}{V_{\mathrm{e}}+dV_{\mathrm{e}}} = \frac{c_{\mathrm{e}}^{\mathrm{avg}}(t)\cdot(V_{\mathrm{e}}-dV_{\mathrm{e}}^{\mathrm{squeeze}})+c_{\mathrm{e}}^{\mathrm{res}}(t)\cdot dV_{\mathrm{e}}^{\mathrm{add}}}{V_{\mathrm{e}}+dV_{\mathrm{EC}}+dV_{\mathrm{e}}^{\mathrm{add}}-dV_{\mathrm{e}}^{\mathrm{squeeze}}}, \tag{S41}$$

$$c_{\mathrm{EC}}(t+dt) = \frac{n_{\mathrm{EC}}+dn_{\mathrm{EC}}}{V_{\mathrm{e}}+dV_{\mathrm{e}}} == \frac{c_{\mathrm{EC}}(t)\cdot(V_{\mathrm{e}}+dV_{\mathrm{EC}}-dV_{\mathrm{e}}^{\mathrm{squeeze}})+c_{\mathrm{EC}}^{\mathrm{res}}(t)\cdot dV_{\mathrm{e}}^{\mathrm{add}}}{V_{\mathrm{e}}+dV_{\mathrm{EC}}+dV_{\mathrm{e}}^{\mathrm{add}}-dV_{\mathrm{e}}^{\mathrm{squeeze}}}, \tag{S42}$$

Notably, we assume that the electrolyte added from the reservoir into the jelly roll changes the Li ions concentration inside the jelly roll at different locations along the electrode direction ($c_{\mathrm{e}}(x,t)$) with the same ratio:

$$c_{\mathrm{e}}(x,t+dt) = \frac{c_{\mathrm{e}}^{\mathrm{avg}}(t+dt)}{c_{\mathrm{e}}^{\mathrm{avg}}(t)} \cdot c_{\mathrm{e}}(x,t). \tag{S43}$$

The illustration of the above volume related quantities needed to describe the solvent consumption model is presented in Supplementary Figure 8.

Depending on the values of $dV_{\mathrm{e}}^{\mathrm{need}}$, $V_{\mathrm{e}}^{\mathrm{res}}$ and A_{cell}, five cases are considered in this work.

- Case-1: The volume of EC consumption ($|dV_{\mathrm{EC}}|$) is <u>larger</u> than that of porosity change ($|dV_{\mathrm{pore}}|$). The jelly roll needs certain amount of electrolyte ($dV_{\mathrm{e}}^{\mathrm{need}}$) to fill the empty space; and the reservoir contains enough electrolyte to fulfil the needs ($V_{\mathrm{e}}^{\mathrm{res}}(t) \geq dV_{\mathrm{e}}^{\mathrm{need}}$). Therefore, $dV_{\mathrm{e}}^{\mathrm{need}}$ of electrolyte transfers from the reservoir to the jelly roll, dry-out does not occur ($R_{\mathrm{dry}} = 1$). The concentration of Li ions and EC inside the jelly roll changes, while those in the reservoir remain the same.

- Case-2: Like Case-1, the volume of EC consumption ($|dV_{\mathrm{EC}}|$) is larger than that of porosity change ($|dV_{\mathrm{pore}}|$). The jelly roll needs certain amount of electrolyte ($dV_{\mathrm{e}}^{\mathrm{need}}$) to fill the empty space. However, the electrolyte volume in the reservoir is not enough to fulfil the needs ($V_{\mathrm{e}}^{\mathrm{res}}(t) < dV_{\mathrm{e}}^{\mathrm{need}}$). Therefore, $V_{\mathrm{e}}^{\mathrm{res}}$ of electrolyte transfers from the reservoir to the jelly roll, dry-out occurs ($R_{\mathrm{dry}} < 1$). The concentration of Li ions and EC inside the jelly roll changes. After the replenishment, the reservoir becomes empty.
- Case-3: The volume of EC consumption ($|dV_{\mathrm{EC}}|$) equals to that of porosity change ($|dV_{\mathrm{pore}}|$). Therefore, no electrolyte moves between the jelly roll and the reservoir ($R_{\mathrm{dry}} = 1$). The concentration of Li ions and EC in the jelly roll and reservoir remain the same.
- Case-4: The volume of EC consumption ($|dV_{\mathrm{EC}}|$) is smaller than to that of porosity change ($|dV_{\mathrm{pore}}|$). Meanwhile, dry out has occurred previously ($V_{\mathrm{e}}^{\mathrm{res}}(t) = 0$, $A_{\mathrm{cell}}(t) < A_{\mathrm{cell,max}}$). Therefore, rewetting occurs, i.e., part of the dry electrode area will become saturated again ($R_{\mathrm{dry}} > 1$). The concentration of Li ions and EC in the jelly roll remain the same.
- Case-5: The volume of EC consumption ($|dV_{\mathrm{EC}}|$) is smaller than to that of porosity change ($|dV_{\mathrm{pore}}|$). Meanwhile, dry out has not occurred previously ($A_{\mathrm{cell}}(t) = A_{\mathrm{cell,max}}$). therefore, certain amount of electrolyte will be squeezed out from the jelly roll to the reservoir ($R_{\mathrm{dry}} = 1$). The concentration of Li ions and EC in the jelly roll remain the same, while those in the reservoir changes.

The above five cases are summarised in Supplementary Table 4.

Supplementary Note 7

Zero-state hysteresis model

PyBaMM has an optional zero-state hysteresis model, in which either the lithiation or delithiation open circuit potential (OCP) is used, depending on the sign of the current:

$$U_{\mathrm{p}}^{\mathrm{OCP}}=\frac{1+\tanh[100\times J]}{2}U_{\mathrm{p,lith}}^{\mathrm{OCP}}+\frac{1-\tanh[100\times J]}{2}U_{\mathrm{p,delith}}^{\mathrm{OCP}} \quad \text{(S44)}$$

$$U_{\mathrm{n}}^{\mathrm{OCP}}=\frac{1-\tanh[100\times J]}{2}U_{\mathrm{n,lith}}^{\mathrm{OCP}}+\frac{1+\tanh[100\times J]}{2}U_{\mathrm{n,delith}}^{\mathrm{OCP}} \quad \text{(S45)}$$

where the applied current density J has units of A/m^2, and is defined as positive for discharge and negative for charge. In our version of PyBaMM, we added an offset of 0.2 A/m^2 to enable the OCP corresponding to discharge to be used when the cell is at rest:

$$U_{\mathrm{p}}^{\mathrm{OCP}}=\frac{1+\tanh[100(J+0.2)]}{2}U_{\mathrm{p,lith}}^{\mathrm{OCP}}+\frac{1-\tanh[100(J+0.2)]}{2}U_{\mathrm{p,delith}}^{\mathrm{OCP}} \quad \text{(S46)}$$

$$U_{\mathrm{n}}^{\mathrm{OCP}}=\frac{1-\tanh[100(J+0.2)]}{2}U_{\mathrm{n,lith}}^{\mathrm{OCP}}+\frac{1+\tanh[100(J+0.2)]}{2}U_{\mathrm{n,delith}}^{\mathrm{OCP}} \quad \text{(S47)}$$

Without this offset, the GITT characterization would not work properly, because when the current is switched off during the rest phase of GITT, the OCP would change from the discharge OCP to the average of the charge and discharge OCPs, which is unphysical. The value of 0.2 A/m^2 is chosen because the magnitude of the current density during the CV charge is always greater than this, so the charge OCP is always used during both CC and CV phases. A comparison between charge/discharge curves with and without the hysteresis model is shown in Supplementary Figure 9.

Supplementary Note 8

Half-cell OCPs and electrode balancing

This cell is chosen because there is a comprehensive set of BOL parameters for the DFN model, published by Chen *et al.*[19] and O'Regan *et al.*[1]. The availability of more recent OCP data enables us to make improvements to the electrode balancing. New half-cell OCP data was provided by Dr. Kieran O'Regan, using the methods of Chen *et al.* The new dataset has two significant advantages over the published one. Firstly, the new data covers the 90-100% stoichiometry range in the graphite-silicon composite, which Chen *et al.* did not. Secondly, raw data for both discharge and charge are available, whereas only the raw data for discharge is included in the PyBaMM parameter set corresponding to Chen *et al.*

The two advantages offered by the updated OCP data are significant because both the interstitial-diffusion limited SEI growth model and the lithium plating model are highly sensitive to the potential of the negative electrode surface, which means an accurate negative electrode OCP is essential to accurate prediction of degradation due to these mechanisms.

The newer (Delithiation, Lithiation) and older (Chen 2020) OCPs are plotted in the top row of Supplementary Figure 10 for comparison.

With the raw data for both charge and discharge OCPs, we can construct analytic OCP functions for both electrodes and both lithiation/delithiation directions:

$$U_{\mathrm{p,lith}}^{\mathrm{OCP}} = -0.7983 \cdot x + 4.513 - 0.03269 \cdot \tanh\big(19.83 \cdot (x - 0.5424)\big) - 18.23 \cdot \tanh\big(14.33 \cdot (x - 0.2771)\big) + 18.05 \cdot \tanh\big(14.46 \cdot (x - 0.2776)\big) \quad \text{(S1)}$$

$$U_{\mathrm{p,delith}}^{\mathrm{OCP}} = -0.7836 \cdot x + 4.513 - 0.03432 \cdot \tanh\big(19.83 \cdot (x - 0.5424)\big) - 19.35 \cdot \tanh\big(14.33 \cdot (x - 0.2771)\big) + 19.17 \cdot \tanh\big(14.45 \cdot (x - 0.2776)\big) \quad \text{(S2)}$$

$$U_{\mathrm{n,lith}}^{\mathrm{OCP}} = 0.5476 \cdot e^{-422.4 \cdot x} + 0.5705 \cdot e^{-36.89 \cdot x} + 0.1336 - 0.04758 \cdot \tanh\big(13.88 \cdot (x - 0.2101)\big) - 0.01761 \cdot \tanh\big(36.2 \cdot (x - 0.5639)\big) - 0.0169 \cdot \tanh\big(11.42 \cdot (x - 1)\big) \quad \text{(S3)}$$

$$U_{\mathrm{n,delith}}^{\mathrm{OCP}} = 1.051 \cdot e^{-26.76 \cdot x} + 0.1916 - 0.05598 \cdot \tanh\big(35.62 \cdot (x - 0.1356)\big) - 0.04483 \cdot \tanh\big(14.64 \cdot (x - 0.2861)\big) - 0.02097 \cdot \tanh\big(26.28 \cdot (x - 0.6183)\big) - 0.02398 \cdot \tanh\big(38.1 \cdot (x - 1)\big) \quad \text{(S4)}$$

where x is the stoichiometry of the electrode. The fitting was performed using MATLAB's Curve Fitting Toolbox. The root mean squared error (RMSE) was less than 8 mV and adjusted R^2 greater than 0.998 for all four fits.

Chen *et al.*[19] did not include data for entropic changes of the two electrodes, but O'Regan *et al.*[1] provided the following functions, which are plotted in the bottom row of Supplementary Figure 10:

$$\frac{\partial U_{\mathrm{p}}}{\partial T} = 0.04006 \cdot e^{-\frac{(x-0.2828)^2}{0.0009855}} - 0.06656 \cdot e^{-\frac{(x-0.8032)^2}{0.02179}} \quad \text{(S5)}$$

$$\frac{\partial U_{\mathrm{n}}}{\partial T} = -0.111 \cdot x + 0.02901 + 0.3562 \cdot e^{-\frac{(x-0.08308)^2}{0.004621}} \quad \text{(S6)}$$

Chen *et al.*[19] and O'Regan *et al.*[1] disagree on the maximum and initial lithium concentrations in each electrode. Neither paper is consistent with the 5Ah total capacity of the cell under (pseudo-)OCP conditions; Chen *et al.*'s parameters result in a larger OCP capacity, while O'Regan *et al.*'s parameters result in a smaller one. We therefore conduct our own electrode balancing for the LG M50 cell.

Inspection of Supplementary Figure 10(a) shows that the negative electrode OCP from O'Regan shows strong agreement with the one reported by Chen *et al.*[19], so we use the stoichiometry limits reported by Chen *et al.*[19] (0.9014 and 0.0279) as a starting point. With all other parameters unchanged, a maximum lithium concentration of 32544 mol/m^3 in the negative electrode results in a capacity of exactly 5 Ah between these limits under OCP conditions.

Upon inspection of Supplementary Figure 10 (b), the agreement between the positive electrode OCPs of Chen *et al*[19] and O'Regan[1] is not as good. The shapes are very similar, but the data from Chen *et al.* is compressed into a smaller stoichiometry range than that from O'Regan *et al.*[1]. To rectify this, new stoichiometry limits of 0.9256 and 0.2411 are calculated using a bisection method and Eq. (S26) to find the stoichiometries that result in the same OCPs as the limits from Chen *et al.*[19] (0.9084 and 0.27) did for the OCP function in that paper. With all other parameters unchanged, a maximum lithium

concentration of 32544 mol/m^3 in the negative electrode results in a (pseudo-)OCP capacity of exactly 5 Ah.

However, Kirkaldy *et al.*[3] measured a smaller capacity of 4.865 Ah. We assume this is due to the cells having degraded due to SEI formation while the cells were in storage. The upper stoichiometry limit is reduced to 0.8771, to account for the difference in capacity.

Supplementary Note 9

Initial SEI thickness

Using the initial capacity loss $Q_{\mathrm{loss,0}} = 5 - 4.865 = 0.135$ Ah, it is possible to calculate the SEI thickness, SEI on cracks thickness and negative porosity at BOL. We assume that the SEI is homogenously distributed on the negative electrode particle surface and crack surface, and each has two layers with the same thickness:

$$L_{\mathrm{SEI,0}}^{\mathrm{inner}} = L_{\mathrm{SEI,0}}^{\mathrm{outer}} = L_{\mathrm{SEIcr,0}}^{\mathrm{inner}} = L_{\mathrm{SEIcr,0}}^{\mathrm{outer}} = 1800 \cdot \frac{Q_{\mathrm{loss,0}}}{F \cdot z_{\mathrm{SEI}}} \cdot \frac{\bar{V}_{\mathrm{SEI}}}{S_{\mathrm{r}} a_n V_{\mathrm{n}}}, \quad \text{(S7)}$$

in which $z_{\mathrm{SEI}} = 2$ is the ratio of lithium moles to SEI moles during the SEI reaction. $\bar{V}_{\mathrm{SEI}}$ [$\mathrm{m^3/mol}$] is the SEI partial molar volume, S_{r} is the roughness ratio, which can be understood as a coefficient to convert the "normal actual surface area of a particle without cracks" to "total area that can growth SEI" when cracks are presented:

$$S_{\mathrm{r}} = 2 l_{\mathrm{cr}} w_{\mathrm{cr}} \rho_{\mathrm{cr}} + 1, \quad \text{(S8)}$$

where $l_{\mathrm{cr}}, w_{\mathrm{cr}}, \rho_{\mathrm{cr}}$ are crack length, crack width and number of cracks per unit area, respectively.

a_{n} is the surface aspect ratio, defined as:

$$a_{\mathrm{n}} = \frac{3\varepsilon_{\mathrm{s,n}}}{R_{\mathrm{n}}}, \quad \text{(S9)}$$

where $\varepsilon_{\mathrm{s,n}}$ and R_{n} are the active material volume fraction and particle radius of the negative electrode.

V_{n} is the volume of the negative electrode (pores included).

Porosity reduction due to such SEI growth is:

$$\Delta\varepsilon = 2 L_{\mathrm{SEI,0}}^{\mathrm{inner}} \cdot S_{\mathrm{r}} a = 3600 \cdot \frac{Q_{\mathrm{loss,0}}}{F \cdot z_{\mathrm{SEI}}} \cdot \frac{\bar{V}_{\mathrm{SEI}}}{V} \quad \text{(S10)}$$

The initial negative porosity from O'Regan *et al.* for a fresh LG M50 cell with a capacity of 5 Ah is 0.25. In our study, the actual initial negative porosity is:

$$\varepsilon_{\mathrm{n,0}} = 0.25 - \Delta\varepsilon \quad \text{(S11)}$$

In our study, we have made $\bar{V}_{\mathrm{SEI}}$ a tuning parameter, which will change both the initial negative electrode porosity and the four SEI thicknesses in Eq. (S7). As an example, for $\bar{V}_{\mathrm{SEI}} = 9.585 \cdot 10^{-5} \frac{\mathrm{m^3}}{\mathrm{mol}}$,

the four initial SEI thicknesses and initial negative electrode porosity will be $1.236 \cdot 10^{-8}$ m and 0.222, respectively.

Supplementary Note 10

Electrolyte parameters

The electrolyte diffusivity, conductivity, cation transference number and thermodynamic factor (Supplementary Figure 11) are based on the EC:EMC 3:7 wt% in $LiPF_6$ from Landesfeind and Gasteiger[2]. We set a saturation limit at 4000 mol/m^3 (4M), assuming that at any salt concentration higher than this value, salt precipitation will happen instantaneously, and the four properties will behave as if the concentration is 4000 mol/m^3 until the concentration drops below this value and the salt dissolves back into the solvent.

$$c_{\mathrm{e}}^{\mathrm{cor}} = \begin{cases} c_{\mathrm{e}}/1000, & c_{\mathrm{e}} < 4000 \\ 4, & c_{\mathrm{e}} \geq 4000 \end{cases} \tag{S12}$$

$$D_{\mathrm{e}} = 10^{-10} \cdot 1010 \cdot e^{1.01 \cdot c_{\mathrm{e}}^{\mathrm{cor}}} \cdot e^{-1560/T} \cdot e^{c_{\mathrm{e}}^{\mathrm{cor}} \cdot (-487)/T} \tag{S13}$$

$$\kappa_{\mathrm{e}} = 0.1 \cdot 0.521 \cdot (1 + (T - 228)) \cdot c_{\mathrm{e}}^{\mathrm{cor}} \cdot \frac{\left(1 - 1.06 \cdot \sqrt{c_{\mathrm{e}}^{\mathrm{cor}}} + 0.8353 \cdot \left(1 - 0.00359 \cdot e^{\frac{1000}{T}}\right) \cdot c_{\mathrm{e}}^{\mathrm{cor}}\right)}{1 + (c_{\mathrm{e}}^{\mathrm{cor}})^4 \cdot \left(0.00148 \cdot e^{\frac{1000}{T}}\right)} \tag{S14}$$

$$\begin{aligned} t_+^0 = -12.8 - 0.612 \cdot c_{\mathrm{e}}^{\mathrm{cor}} + 0.0821 \cdot T + 0.904 \cdot (c_{\mathrm{e}}^{\mathrm{cor}})^2 + 0.0318 \cdot c_{\mathrm{e}}^{\mathrm{cor}} \cdot T \\ - 1.27 \cdot 10^{-4} \cdot T^2 + 0.0175 \cdot (c_{\mathrm{e}}^{\mathrm{cor}})^3 - 3.12 \cdot 10^{-3} \cdot (c_{\mathrm{e}}^{\mathrm{cor}})^2 \cdot T \\ - 3.96 \cdot 10^{-5} \cdot c_{\mathrm{e}}^{\mathrm{cor}} \cdot T^2 \end{aligned} \tag{S15}$$

$$\begin{aligned} \chi = 25.7 - 45.1 \cdot c_{\mathrm{e}}^{\mathrm{cor}} - 0.177 \cdot T + 1.94 \cdot (c_{\mathrm{e}}^{\mathrm{cor}})^2 + 0.295 \cdot c_{\mathrm{e}}^{\mathrm{cor}} \cdot T + 3.08 \cdot 10^{-4} \\ \cdot T^2 + 0.259 \cdot (c_{\mathrm{e}}^{\mathrm{cor}})^3 - 9.46 \cdot 10^{-3} \cdot (c_{\mathrm{e}}^{\mathrm{cor}})^2 \cdot T - 4.54 \cdot 10^{-4} \\ \cdot c_{\mathrm{e}}^{\mathrm{cor}} \cdot T^2 \end{aligned} \tag{S16}$$

Supplementary Note 11

Other beginning of life parameters

The remaining model parameters for BOL are unchanged from those reported by O'Regan *et al.*[1]. All beginning of life parameters are listed in Supplementary Table 6, where modified parameters are denoted by an asterisk (*).

Solid-phase diffusivity for negative and positive electrode (Supplementary Figure 12) follows:

$$\log_{10}\left(D_s^{\mathrm{ref}}/R_{\mathrm{cor}}\right) = a_0 \cdot x + b_0 + a_1 \cdot e^{-\frac{(x-b_1)^2}{c_1}} + a_2 \cdot e^{-\frac{(x-b_2)^2}{c_2}} + +a_3 \cdot e^{-\frac{(x-b_3)^2}{c_3}} + a_4 \cdot e^{-\frac{(x-b_4)^2}{c_4}} \quad \text{(S17)}$$

$$D_s = D_s^{\mathrm{ref}} \cdot e^{-\frac{E_{\mathrm{act}}}{R}\cdot\left(\frac{1}{T}-\frac{1}{T_{\mathrm{ref}}}\right)} \quad \text{(S18)}$$

The positive electrode electronic conductivity is:

$$\sigma_{\mathrm{s,p}} = 0.8473 \cdot e^{-\frac{3500}{R}\cdot\left(\frac{1}{T}-\frac{1}{298.15}\right)} \quad \text{(S19)}$$

The specific heat capacity of the wet electrode $\bar{\theta}$ is calculated by combining those of each bulk materials in the electrode:

$$\bar{\theta} = \rho_{\mathrm{s}} \cdot C_{\mathrm{p,s}} \cdot \varepsilon_s + \rho_{\mathrm{l}} \cdot C_{\mathrm{p,l}} \cdot \varepsilon_l, \quad \text{(S20)}$$

where ρ, C_{p}, and ε are the density, gravimetric heat capacity and volume fraction for the porous electrode/separator and electrolyte, respectively. The specific heat capacities of the solid parts (electrode and separator) are:

$$C_{\mathrm{p,pos}} = -0.0008414 \cdot T^3 + 0.7892 \cdot T^2 - 241.3 \cdot T + 2.508 \cdot 10^4 \quad \text{(S21)}$$

$$C_{\mathrm{p,neg}} = 0.0004932 \cdot T^3 - 0.491 \cdot T^2 + 169.4 \cdot T - 1.897 \cdot 10^4 \quad \text{(S22)}$$

$$C_{\mathrm{p,sep}} = 0.001494 \cdot T^3 - 1.444 \cdot T^2 + 475.5 \cdot T - 5.13 \cdot 10^4 \quad \text{(S23)}$$

Note *T* in the above equations is in Kelvin.

The thermal conductivities of wet electrodes are:

$$\lambda_{\mathrm{pos}} = 2.063 \cdot 10^{-5} \cdot T^2 - 0.01127 \cdot T + 2.331 \quad \text{(S24)}$$

$$\lambda_{\mathrm{neg}} = -2.61 \cdot 10^{-4} \cdot T^2 + 0.1726 \cdot T - 24.49 \quad \text{(S25)}$$

Exchange current densities for intercalation of both electrodes are:

$$j_{0,\mathrm{p}}^{\mathrm{int}} = 5.028 \cdot e^{-\frac{2.401 \cdot 10^4}{R} \cdot \left(\frac{1}{T} - \frac{1}{298.15}\right)} \cdot \left(\frac{c_{\mathrm{e}}}{\langle c_{\mathrm{e}} \rangle}\right)^{0.57} \cdot \left(\frac{c_{\mathrm{s,suf}}}{c_{\mathrm{s,max}}}\right)^{0.43} \cdot \left(1 - \frac{c_{\mathrm{s,suf}}}{c_{\mathrm{s,max}}}\right)^{0.57} \quad \text{(S26)}$$

$$j_{0,\mathrm{n}}^{\mathrm{int}} = 2.668 \cdot e^{-\frac{4 \cdot 10^4}{R} \cdot \left(\frac{1}{T} - \frac{1}{298.15}\right)} \cdot \left(\frac{c_{\mathrm{e}}}{\langle c_{\mathrm{e}} \rangle}\right)^{0.208} \cdot \left(\frac{c_{\mathrm{s,suf}}}{c_{\mathrm{s,max}}}\right)^{0.792} \cdot \left(1 - \frac{c_{\mathrm{s,suf}}}{c_{\mathrm{s,max}}}\right)^{0.208} \quad \text{(S27)}$$

Supplementary Note 12

Calculation of degradation modes (DMs)

DM analysis is a well-established method used in diagnosing the degradation of LIBs[20-22]. DMs can be calculated from both experiment data by OCP fitting[23] or directly from internal variables from physics-based models[24]. Here we mainly adapt the formulas from [24]. The OCP fitting method reconstructs the full cell voltage at low current rate (in this work, C/10) by combing the OCPs of the two electrodes:

$$\tilde{V} = U_{\mathrm{p}}(y) - U_{\mathrm{n}}(x), \tag{S28}$$

where $\tilde{V}$ is the reconstructed full cell voltage, y and x are the electrode stoichiometries. During discharge, the electrode stoichiometries can be calculated as

$$x(t) = x_{100} - \frac{Q(t)}{C_{\mathrm{n}}}, \tag{S29}$$

$$y(t) = y_{100} + \frac{Q(t)}{C_{\mathrm{p}}}, \tag{S30}$$

where $Q(t)$ $[\mathrm{A \cdot h}]$ is the discharge capacity calculated during the discharge process by coulomb counting. x_{100} and y_{100} are the electrode stoichiometries at the upper cell voltage limit V_{max}. C_{n} and C_{p} $[\mathrm{A \cdot h}]$ are the capacities of the negative and positive electrode, respectively, which can be linked to the experimentally measured cell capacity C $[\mathrm{A \cdot h}]$ through

$$C = C_{\mathrm{n}}(x_{100} - x_0) = C_{\mathrm{p}}(y_0 - y_{100}), \tag{S31}$$

where x_0 and y_0 are the electrode stoichiometries at the lower cell voltage limit V_{min}. The RMSE between the reconstructed voltage and the measured voltage is

$$\mathrm{RMSE} = \frac{1}{n}\sum_{t=t_0}^{t_n}\left[V(t) - \tilde{V}(t)\right]^2. \tag{S32}$$

With above equations, the four parameters x_{100}, y_{100}, C_{n}, C_{p} can be determined by minimizing the RMSE using optimization methods. Then, x_0 and y_0 can also be obtained via Eq. (S78). Alternatively, we can also choose to identify x_{100}, y_{100}, x_0 and y_0 then calculate C_{n} and C_{p}. The total amount of intercalated lithium in the cell n_{Li} [mol] is

$$n_{\mathrm{Li}} = \frac{3600}{F}\left(x_{100} \cdot C_{\mathrm{n}} + y_{100} \cdot C_{\mathrm{p}}\right). \tag{S33}$$

The DMs can now be calculated as:

$$\mathrm{LAM_{NE}} = \left(1 - \frac{C_\mathrm{n}}{C_\mathrm{n,0}}\right) \cdot 100\%, \tag{S34}$$

$$\mathrm{LAM_{PE}} = \left(1 - \frac{C_\mathrm{p}}{C_\mathrm{p,0}}\right) \cdot 100\%, \tag{S35}$$

$$\mathrm{LLI} = \left(1 - \frac{n_\mathrm{Li}}{n_\mathrm{Li,0}}\right) \cdot 100\%, \tag{S36}$$

The subscript 0 in $C_\mathrm{n,0}$, $C_\mathrm{p,0}$ and $n_\mathrm{Li,0}$ represents the results identified from the first RPT (BOL). For physics-based models, the calculation of DMs is easier and more straightforward, as the model intrinsically track these internal variables.

Supplementary Note 13

Parametrization results

To investigate such competing effects, we have chosen three values of $E_{\mathrm{act}}^{\mathrm{SEI}}$ (5×10^2, 5×10^3, 5×10^4 J/mol) and of $E_{\mathrm{act}}^{\mathrm{D_{s,n}}}$ (2×10^4, 4×10^4, 6×10^4 J/mol), respectively, resulting in 9 combinations. The resulting temperature dependencies are presented in Supplementary Figure 13.

For the *SEI only* model, three combinations of ($E_{\mathrm{act}}^{\mathrm{SEI}}$, $E_{\mathrm{act}}^{\mathrm{D_{s,n}}}$) can reproduce the experimentally observed temperature dependency (Supplementary Figure 13 (a)), namely (5×10^3, 2×10^4) J/mol, (5×10^3, 4×10^4) J/mol, (1×10^4, 6×10^4) J/mol. For the *SEI + Dry out* model, LLI has two sources (Supplementary Figure 14): lithium locked in the SEI and lithium trapped in the electrode host material that has become inactive due to dry-out induced LAM. As presented in Supplementary Figure 14, LLI due to dry-out is almost twice that the amount due to SEI under all three temperatures. However, dry out is a direct result of the SEI growth. Therefore, the total LLI and total capacity loss of the *SEI + Dry out* model follows the same temperature dependency (Supplementary Figure 13 (b)) as that of the *SEI only* model. For the parameters used of the *5 coupled* model, LLI of has 4 sources: SEI, SEI on cracks, lithium plating and LAM, among which LAM accounts for the largest portion (Supplementary Figure 15). The LAM induced LLI can be further decomposed into 2 parts, LLI induced by the dry-out induced, and that induced by the stress-driven LAM. As presented in Supplementary Figures 13 and 15, the temperature dependencies of both LLI and LAM are mainly caused by the dry-out induced LLI. However, the temperature dependency of stress-driven LAM is caused solely by $E_{\mathrm{act}}^{\mathrm{D_{s,n}}}$, i.e., a higher value of $E_{\mathrm{act}}^{\mathrm{D_{s,n}}}$ gives a lower solid diffusivity under low temperatures, and therefore a higher stress-driven LAM. As a result, only two combinations of ($E_{\mathrm{act}}^{\mathrm{SEI}}$, $E_{\mathrm{act}}^{\mathrm{D_{s,n}}}$) can reproduce the desirable temperature dependency, namely (5×10^3, 2×10^4) J/mol and (5×10^3, 4×10^4) J/mol. In summary, we have found that all three models in this work can reproduce the experimentally observed temperature dependent capacity fade, even for different reasons.

Supplementary References

(1) O'Regan, K.; Brosa Planella, F.; Widanage, W. D.; Kendrick, E. Thermal-electrochemical parameters of a high energy lithium-ion cylindrical battery. *Electrochimica Acta* **2022**, *425*. DOI: 10.1016/j.electacta.2022.140700.

(2) Landesfeind, J.; Gasteiger, H. A. Temperature and Concentration Dependence of the Ionic Transport Properties of Lithium-Ion Battery Electrolytes. *Journal of The Electrochemical Society* **2019**, *166* (14), A3079-A3097. DOI: 10.1149/2.0571912jes.

(3) Kirkaldy, N.; Samieian, M. A.; Offer, G. J.; Marinescu, M.; Patel, Y. Lithium-ion battery degradation: Comprehensive cycle ageing data and analysis for commercial 21700 cells. *Journal of Power Sources* **2024**, *603*. DOI: 10.1016/j.jpowsour.2024.234185.

(4) O'Kane, S. E. J.; Ai, W. L.; Madabattula, G.; Alonso-Alvarez, D.; Timms, R.; Sulzer, V.; Edge, J. S.; Wu, B.; Offer, G. J.; Marinescu, M. Lithium-ion battery degradation: how to model it. *Physical Chemistry Chemical Physics* **2022**, *24* (13), 7909-7922, Article. DOI: 10.1039/d2cp00417h.

(5) Zhu, J. G.; Knapp, M.; Sorensen, D. R.; Heere, M.; Darma, M. S. D.; Muller, M.; Mereacre, L.; Dai, H. F.; Senyshyn, A.; Wei, X. Z.; et al. Investigation of capacity fade for 18650-type lithium-ion batteries cycled in different state of charge (SoC) ranges. *Journal of Power Sources* **2021**, *489*, 12, Article. DOI: 10.1016/j.jpowsour.2020.229422.

(6) Ecker, M.; Nieto, N.; Kabitz, S.; Schmalstieg, J.; Blanke, H.; Warnecke, A.; Sauer, D. U. Calendar and cycle life study of Li(NiMnCo)O-2-based 18650 lithiumion batteries. *Journal of Power Sources* **2014**, *248*, 839-851, Article. DOI: 10.1016/j.jpowsour.2013.09.143.

(7) Käbitz, S.; Gerschler, J. B.; Ecker, M.; Yurdagel, Y.; Emmermacher, B.; André, D.; Mitsch, T.; Sauer, D. U. Cycle and calendar life study of a graphite|LiNi_{1/3}Mn_{1/3}Co_{1/3}O₂ Li-ion high energy system. Part A: Full cell characterization. *Journal of Power Sources* **2013**, *239*, 572-583, Article. DOI: 10.1016/j.jpowsour.2013.03.045.

(8) Single, F.; Latz, A.; Horstmann, B. Identifying the Mechanism of Continued Growth of the Solid-Electrolyte Interphase. *Chemsuschem* **2018**, *11* (12), 1950-1955, Article. DOI: 10.1002/cssc.201800077.

(9) von Kolzenberg, L.; Latz, A.; Horstmann, B. Solid-Electrolyte Interphase During Battery Cycling: Theory of Growth Regimes. *ChemSusChem* **2020**, *13* (15), 3901-3910. DOI: 10.1002/cssc.202000867.

(10) Zhang, X. C.; Shyy, W.; Sastry, A. M. Numerical simulation of intercalation-induced stress in Li-ion battery electrode particles. *Journal of the Electrochemical Society* **2007**, *154* (10), A910-A916, Article. DOI: 10.1149/1.2759840.

(11) Deshpande, R.; Verbrugge, M.; Cheng, Y. T.; Wang, J.; Liu, P. Battery Cycle Life Prediction with Coupled Chemical Degradation and Fatigue Mechanics. *Journal of the Electrochemical Society* **2012**, *159* (10), A1730-A1738, Article. DOI: 10.1149/2.049210jes.

(12) Laresgoiti, I.; Kabitz, S.; Ecker, M.; Sauer, D. U. Modeling mechanical degradation in lithium ion batteries during cycling: Solid electrolyte interphase fracture. *Journal of Power Sources* **2015**, *300*, 112-122, Article. DOI: 10.1016/j.jpowsour.2015.09.033.

(13) Reniers, J. M.; Mulder, G.; Howey, D. A. Review and Performance Comparison of Mechanical-Chemical Degradation Models for Lithium-Ion Batteries. *Journal of the Electrochemical Society* **2019**, *166* (14), A3189-A3200, Review. DOI: 10.1149/2.0281914jes.

(14) Li, R.; O'Kane, S.; Marinescu, M.; Offer, G. J. Modelling solvent consumption from SEI layer growth in lithium-ion batteries. *Journal of the Electrochemical Society* **2022**, *169* (6), 14, Article. DOI: 10.1149/1945-7111/ac6f84.

(15) Li, R.; O'Kane, S.; Wang, A.; Jung, T.; Kirkaldy, N.; Marinescu, M.; Monroe, C. W.; Offer, G. J. Lithium-ion battery performance model including solvent segregation effects. *arXiv* **2023**, *[arXiv:2311.05467v1]*. DOI: 10.48550/arXiv.2311.05467.

(16) Bogle, X.; Vazquez, R.; Greenbaum, S.; Cresce, A. V.; Xu, K. Understanding Li+-Solvent Interaction in Nonaqueous Carbonate Electrolytes with O-17 NMR. *Journal of Physical Chemistry Letters* **2013**, *4* (10), 1664-1668, Article. DOI: 10.1021/jz400661k.

(17) Xu, K.; Lam, Y. F.; Zhang, S. S.; Jow, T. R.; Curtis, T. B. Solvation sheath of Li+ in nonaqueous electrolytes and its implication of graphite/electrolyte interface chemistry. *Journal of Physical Chemistry C* **2007**, *111* (20), 7411-7421, Article. DOI: 10.1021/jp068691u.
(18) Cresce, A. V.; Borodin, O.; Xu, K. Correlating Li+ Solvation Sheath Structure with Interphasial Chemistry on Graphite. *Journal of Physical Chemistry C* **2012**, *116* (50), 26111-26117, Article. DOI: 10.1021/jp303610t.
(19) Chen, C. H.; Planella, F. B.; O'Regan, K.; Gastol, D.; Widanage, W. D.; Kendrick, E. Development of Experimental Techniques for Parameterization of Multi-scale Lithium-ion Battery Models. *Journal of the Electrochemical Society* **2020**, *167* (8), 22, Article. DOI: 10.1149/1945-7111/ab9050.
(20) Dubarry, M.; Truchot, C.; Liaw, B. Y. Synthesize battery degradation modes via a diagnostic and prognostic model. *Journal of Power Sources* **2012**, *219*, 204-216, Article. DOI: 10.1016/j.jpowsour.2012.07.016.
(21) Dubarry, M.; Truchot, C.; Liaw, B. Y. Cell degradation in commercial LiFePO4 cells with high-power and high-energy designs. *Journal of Power Sources* **2014**, *258*, 408-419, Article. DOI: 10.1016/j.jpowsour.2014.02.052.
(22) Baure, G.; Dubarry, M. Synthetic vs. Real Driving Cycles: A Comparison of Electric Vehicle Battery Degradation. *Batteries-Basel* **2019**, *5* (2), 15, Article. DOI: 10.3390/batteries5020042.
(23) Han, X. B.; Ouyang, M. G.; Lu, L. G.; Li, J. Q.; Zheng, Y. J.; Li, Z. A comparative study of commercial lithium ion battery cycle life in electrical vehicle: Aging mechanism identification. *Journal of Power Sources* **2014**, *251*, 38-54, Article. DOI: 10.1016/j.jpowsour.2013.11.029.
(24) Sulzer, V.; Mohtat, P.; Pannala, S.; Siegel, J. B.; Stefanopoulou, A. G. Accelerated Battery Lifetime Simulations Using Adaptive Inter-Cycle Extrapolation Algorithm. *Journal of the Electrochemical Society* **2021**, *168* (12), 12, Article. DOI: 10.1149/1945-7111/ac3e48.
